# Microphase separation produces interfacial environment within diblock biomolecular condensates

**DOI:** 10.7554/eLife.90750

**Published:** 2025-03-26

**Authors:** Andrew P Latham, Longchen Zhu, Dina A Sharon, Songtao Ye, Adam P Willard, Xin Zhang, Bin Zhang

**Affiliations:** 1 Department of Chemistry, Massachusetts Institute of Technology Cambridge United States; 2 Department of Chemistry, School of Science and Research Center for Industries of the Future, Westlake University Hangzhou China; 3 Westlake Laboratory of Life Sciences and Biomedicine Hangzhou China; University of Cambridge United Kingdom; Boston University United States

**Keywords:** biomolecular condensates, molecular dynamics, multiscale simulations, hydrophobicity, None

## Abstract

The phase separation of intrinsically disordered proteins is emerging as an important mechanism for cellular organization. However, efforts to connect protein sequences to the physical properties of condensates, that is, the molecular grammar, are hampered by a lack of effective approaches for probing high-resolution structural details. Using a combination of multiscale simulations and fluorescence lifetime imaging microscopy experiments, we systematically explored a series of systems consisting of diblock elastin-like polypeptides (ELPs). The simulations succeeded in reproducing the variation of condensate stability upon amino acid substitution and revealed different microenvironments within a single condensate, which we verified with environmentally sensitive fluorophores. The interspersion of hydrophilic and hydrophobic residues and a lack of secondary structure formation result in an interfacial environment, which explains both the strong correlation between ELP condensate stability and interfacial hydrophobicity scales, as well as the prevalence of protein-water hydrogen bonds. Our study uncovers new mechanisms for condensate stability and organization that may be broadly applicable.

## Introduction

Biological condensates are found in both the cytosol (Brangwynne et al., 2009; Folkmann et al., 2021; Patel et al., 2015; Ma and Mayr, 2018) and nucleus, (Feric et al., 2016; Larson et al., 2017; Strom et al., 2017; Sabari et al., 2018; Leicher et al., 2022; Latham and Zhang, 2022b), playing essential roles in a variety of cellular processes (Banani et al., 2017; Uversky, 2017) from stress response (Riback et al., 2017) to genome organization (Lin et al., 2021; Sabari et al., 2020). Similar to membrane-bound organelles, they assemble a collection of molecules to raise the efficiency of sophisticated tasks. The lack of a membrane barrier allows fast material exchange between condensates and the cellular environment, rendering the molecular composition and stability of condensates more prone to regulations by external signals (Hnisz et al., 2017; Riback et al., 2020; Klein et al., 2020).

Intrinsically disordered proteins (IDPs) that promote multivalent, promiscuous interactions are key drivers of condensate formation (Hyman et al., 2014; Boeynaems et al., 2018; Abyzov et al., 2022). Multiple mechanisms, including electrostatic, cation-π, π-π, hydrogen bonding, and hydrophobic interactions, contribute to the affinity among various chemical groups (Dignon et al., 2020; Das et al., 2020; Murthy et al., 2021). Above a threshold concentration, as predicted by the Flory–Huggins theory (Flory, 1942), interactions among IDPs can drive liquid-liquid phase separation to produce a highly concentrated phase that nevertheless remains dynamic. The simplicity of theory, while insightful, may prove insufficient for a comprehensive understanding of biological condensates.

Much remains to be learned regarding the connection between amino acid sequences and protein phase behaviors, or the so-called ‘molecular grammar’ of protein condensates (Schuster et al., 2021; Choi et al., 2020; Wang et al., 2018; Schneider et al., 2019; Elbaum-Garfinkle et al., 2015; Kilgore and Young, 2022). These systems often exhibit complex viscoelastic behaviors and substructures with a layered organization (Latham and Zhang, 2022a; Mittag and Pappu, 2022; Kar et al., 2022; Wu and King, 2023), defying the mean-field assumption in the Flory–Huggins theory. Several more advanced theories have been introduced to better account for long-lived structural features that might persist in the polymer network. The sticker and spacer model (Tanaka, 1989; Semenov and Rubinstein, 1998) has been adopted by Pappu and coworkers to account for strong and specific interactions that might form physical cross-links among protein molecules (Harmon et al., 2017). In the meantime, the block copolymer theory may explain the microphase separation that can lead to layered structures (Leibler, 1980; Bates and Fredrickson, 1990; Matsen and Schick, 1994; Matsen and Bates, 1996; Grason, 2006; Swann and Topham, 2010; Shi, 2021). Because of their inherent assumptions, different theories are more appropriate for some systems but not others. High-resolution structural characterizations of condensates could offer further insight into their organizational principles and the applicable theories.

Further decoding the condensate grammar could also benefit from studies of simpler systems. Natural proteins often utilize highly complex amino acid sequences, rendering the attribution of an individual residue’s contribution to collective phenomena challenging. On the other hand, elastin-like polypeptides (ELPs), which are composed of pentapeptide repeats of valine-proline-glycine-X-glycine, serve as excellent models for studying protein phase behaviors. ELPs undergo phase separation upon heating with a lower critical solution temperature (LCST) (Urry, 1997; Rauscher and Pomès, 2017; Baul et al., 2020; Cinar et al., 2019; Dignon et al., 2019). Importantly, the guest residue X can be modified to any amino acid except proline with modern engineering approaches (McDaniel et al., 2010), enabling systematic characterization of the impact on condensate stability and organization upon introducing specific residues.

We combine multiscale simulations with fluorescence lifetime imaging microscopy (FLIM) to probe condensates formed by diblock ELPs and investigate the contribution of amino acid composition to condensate stability. The simulation approach allows the sampling of large-scale conformational rearrangements while providing atomistic resolution to quantify the solvation environment of individual residues. It succeeds in reproducing the stability of condensates and reveals the formation of tubular network phases similar to gyroids. Such structures deviate from weak micelles that have been proposed for diblock ELPs in solution and result in heterogeneous microenvironments within the condensate, which we verify in vitro with FLIM. In addition, we find that the condensate stability exhibits a striking correlation with a hydrophobicity scale derived from interfacial transfer free energy, supporting an interfacial microenvironment of the condensate interior. The chemical specificity of the microenvironment is dictated by the peptide sequence that prevents a complete microphase separation between hydrophobic and hydrophilic groups. As a result, all condensates remain highly solvated after phase separation, producing water molecules that maintain hydrogen bonding with the exposed peptide backbone that persists even in residues with hydrophobic side chains. Our study takes a significant stride at connecting amino acid sequences with the structural and physical properties of biological condensates.

## Results

### Multiscale simulations of diblock ELP condensates

**Figure 1.**
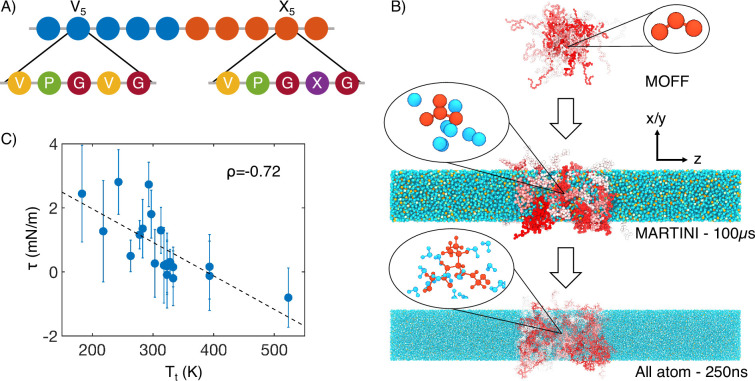
Multiscale simulations enable thermodynamic and structural characterization of elastin-like polypeptide (ELP) condensates. (**A**) Illustration of the sequence for the simulated diblock ELPs that consist of five-amino acid repeats, where X is substituted with a guest amino acid. (**B**) Overview of the three-step multiscale simulation approach that gradually increases the model resolution. Simulations of V5L5 were used to produce the example configurations at each step. Peptides are shaded red-white, while water molecules, chlorine, and sodium are colored in blue, green, and orange, respectively. Inserts of a G-V-G repeat and surrounding water molecules are shown to indicate the resolution of each model. (**C**) Correlation between the simulated surface tension (τ) of 20 ELP condensates and the transition temperatures ($T_{t}$) of related systems (Urry, 1997) ρ is the Pearson correlation coefficient between the two data sets, and the dashed line is the best fit between simulation and experimental data. Error bars represent the SD of estimates from five independent time windows.

Decoding the condensate grammar necessitates connecting protein sequences with collective physical properties. ELPs stand out because of their sequence simplicity and amenability for biological engineering, allowing systematic exploration and precise attribution of amino acid contributions. We focus on diblock ELPs of sequence (V-P-G-V-G)*_n_*-(V-P-G-X-G)_*n*_, which we abbreviate as V_*n*_X_*n*_ (Figure 1A). The guest residue in the first block is set as valine to promote phase separation, and we explore 20 systems in which every natural amino acid is substituted into the X position in the second block.

We adopt a multiscale approach to balance accuracy and efficiency for simulating ELP condensates (Figure 1B). The first stage of this strategy is to simulate condensate formation using a coarse-grained force field, MOFF (Latham and Zhang, 2021; Latham and Zhang, 2019; Latham and Zhang, 2020; Latham and Zhang, 2022c), with one bead per amino acid and implicit solvation. These simulations are highly efficient for system relaxation and promoting large-scale conformational rearrangements that occur over slow timescales. We further converted these α-carbon-based models to approximately four heavy atoms per bead resolution and introduced explicit water and ions for simulations with the MARTINI force field (Marrink et al., 2007). The latest version of this force field (MARTINI3) provides a more balanced set of parameters for protein-protein interactions and has been applied to study biological condensates (Souza et al., 2021; Thomasen et al., 2022; Benayad et al., 2021; Tsanai et al., 2021). These simulations further relax the protein configurations and the partition of water and counterions in condensed and dilute phases. Finally, we carried out explicit solvent all-atom simulations for 250 ns to produce an accurate characterization of the chemical environment of the condensates.

Utilizing the simulated condensate conformations, we computed various quantities to benchmark against experimental measurements. While the critical temperature has been widely used as a measure for condensate stability, determining it computationally is expensive. As an alternative, we computed the surface tension, τ, using 100-μs-long MARTINI simulations performed with the NP_N_AT ensemble (Zhang et al., 1995). As detailed in the ‘Supplemental theory’ in Appendix 1, an inverse relationship is expected between τ and the critical temperature, $T_{c}$, for systems exhibiting LCSTs. We further approximate $T_{c}$ with the transition temperatures ($T_{t}$) of ELP sequences (Urry, 1997), which are the temperatures at which ELPs undergo an LCST transition at a specified solution condition. $T_{t}$ was shown to be linearly proportional to $T_{C}$ (McDaniel et al., 2013; Meyer and Chilkoti, 2004). As expected, a negative correlation can be readily seen between computed surface tension and experimental $T_{t}$ (Figure 1C). This observed negative correlation between $T_{t}$ and τ supports the simulation approach’s accuracy in reproducing the sequence-dependent changes in ELP phase behavior.

We note that the experimental values were determined using different ELP sequences from those simulated here, and the relation between the surface tension and $T_{c}$ is likely nonlinear (Roe, 1975), both contributing to the imperfect agreement. In another study (Ye et al., 2024), we adopted the same multiscale approach to determine the dielectric constant for several ELP condensates. The simulated values agree well with those determined using FLIM experiments.

### Microphase separation of ELP condensates

Upon validating the accuracy of the simulation protocol for reproducing collective properties of ELP condensates, we examined their structural organization. Hassouneh et al. argued that, in dilute solutions, ELP diblocks self-assemble into the so-called weak spherical micelles with dense cores and almost unstretched coronas (Hassouneh et al., 2015). At phase separation conditions, whether similar structural features are preserved in condensates remains unclear.

**Figure 2.**
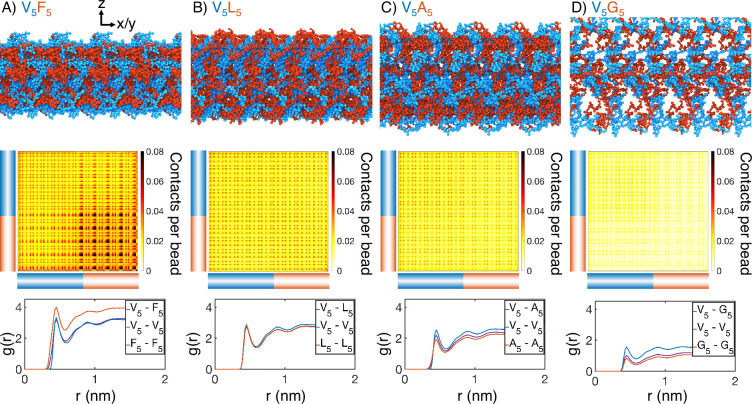
Internal organization of elastin-like polypeptide (ELP) condensates for (**A**) V_5_F_5_, (**B**) V_5_L_5_, (**C**) V_5_A_5_, and (**D**) V_5_G_5_. The uppermost panels present representative configurations from MARTINI simulations for each system. Periodic images along the x and y dimensions are shown for clarity, and the condensate-water interface is perpendicular to the z-axis. Only proteins are shown, with the X- and V-substituted halves of the peptides shown in red and blue, respectively. The central panels show contact maps between amino acids from different peptides. The blue and red bars indicate the V- and X-substituted half of the peptides. The lower panels plot the radial distribution functions, g(r), for amino acids only from the V-substituted half of the peptides (V_5_-V_5_), only from the X-substituted half of the peptides (X_5_-X_5_), and between the two halves (V_5_-X_5_). We limited the calculations to amino acid pairs from different peptides.

We found that most ELP condensates undergo a microphase separation. Representative configurations for four typical systems from MARTINI simulations are shown in the top panels of Figure 2, with the V blocks in blue and X in red. Overall, the microphase separation manages to orient the more hydrophobic blocks toward the condensate interior as opposed to the interface. The relative density of guest blocks near the center of the condensate positively correlates with their hydrophobicity, as can be seen in both MARTINI (Figure 2—figure supplement 1) and all-atom (Figure 2—figure supplement 2) simulations.

**Figure 2—figure supplement 1.**
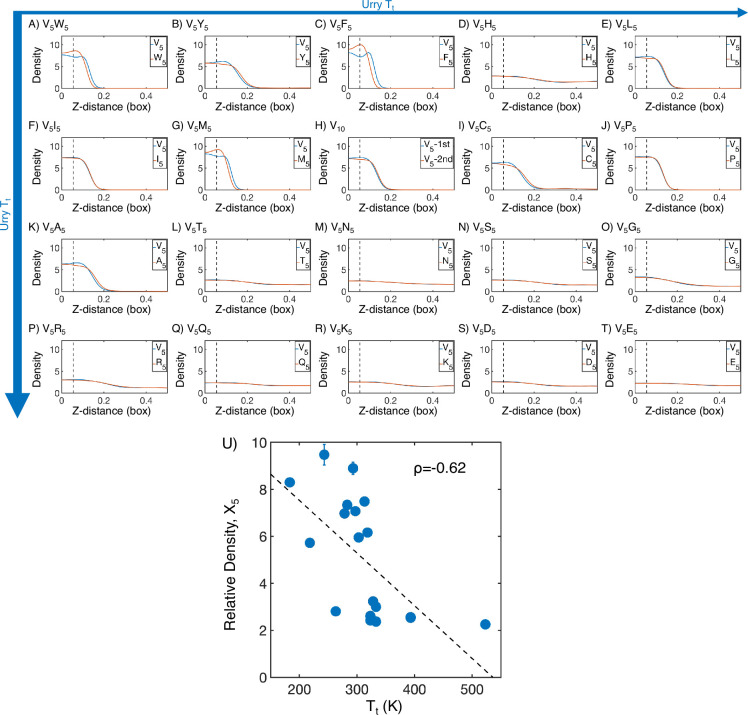
Mass density profiles of elastin-like polypeptide (ELP) condensates from MARTINI simulations. (**A–T**) The relative mass density along the *Z*-distance from the condensate center is shown for the V-substituted and X-substituted halves of each condensate. The *Z*-axis is defined as the direction perpendicular to the condensate-water interface. The dashed line represents a *Z*-distance of 0.06 box lengths away from the condensate center. Average density values below this threshold are used for correlation analysis in (**U**). Proteins are arranged by increasing $T_{t}$ (decreasing hydrophobicity) from Urry, 1997 with (**A**, V_5_W_5_) being the most hydrophobic and (**T**, V_5_E_5_) being the least hydrophobic. (**U**) Correlation between the mass fraction of the X_5_ half of the condensate and transition temperature ($T_{t}$) from Urry, 1997. ρ is the Pearson correlation coefficient between the two data sets, and the dashed diagonal line is the best-fit line. Error bars represent SDs of the mean taken over six equally spaced box length intervals.

**Figure 2—figure supplement 2.**
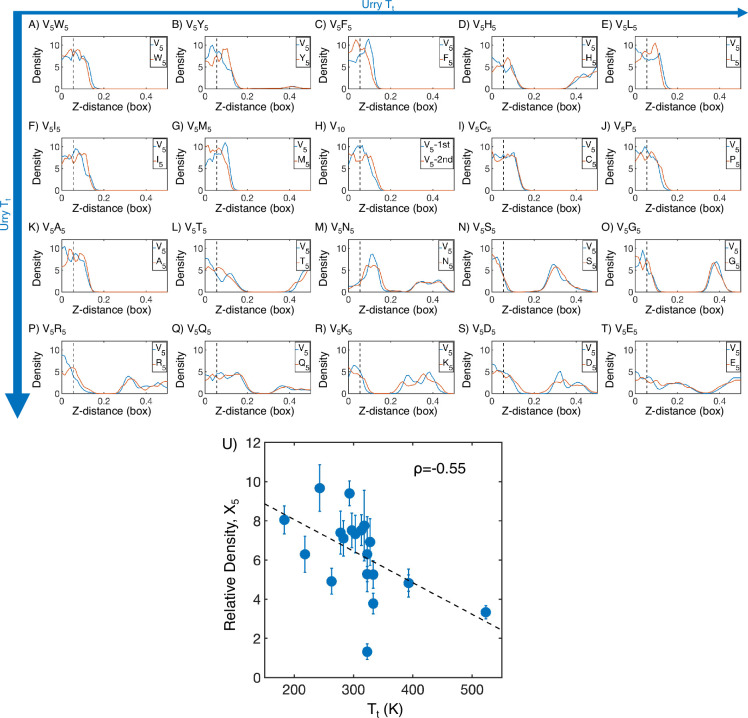
Mass density profiles of elastin-like polypeptide (ELP) condensates from all-atom simulations. (**A–T**) The relative mass density along the *Z*-distance from the condensate center is shown for the V-substituted and X-substituted halves of each condensate. The *Z*-axis is defined as the direction perpendicular to the condensate-water interface. The dashed line represents a *Z*-distance of 0.06 box lengths away from the condensate center. Average density values below this threshold are used for correlation analysis in (**U**). Proteins are arranged by increasing $T_{t}$ (decreasing hydrophobicity) from , Urry, 1997 with (**A**, V_5_W_5_) being the most hydrophobic and (**T**, V_5_E_5_) being the least hydrophobic. (**U**) Correlation between the mass fraction of the X_5_ half of the condensate and transition temperature ($T_{t}$) from Urry, 1997. ρ is the Pearson correlation coefficient between the two data sets, and the dashed diagonal line is the best-fit line. Error bars represent SDs of the mean taken over six equally spaced box length intervals.

**Figure 2—figure supplement 3.**
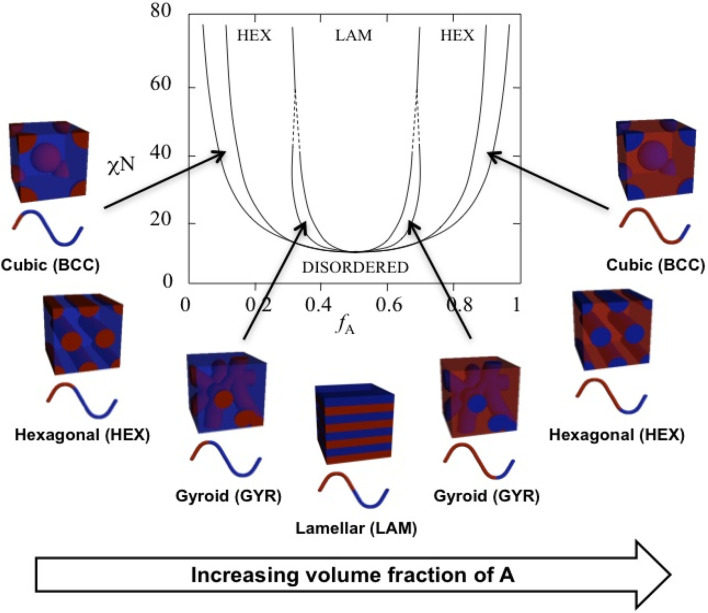
Theoretical phase diagram (Matsen and Bates, 1996) and corresponding morphologies for diblock copolymers. The phases are labeled as body centered cubic (BCC), hexagonal cylinders (HEX), gyroid (GYR), and lamellar (LAM). $f_{A}$ is the volume fraction of a single polymer block, denoted A, χ is the Flory–Huggins interaction parameter, and N is the total degree of polymerization. Reproduced from Figure 3 from Swann and Topham, 2010 (CC BY 4.0).

**Figure 2—figure supplement 4.**
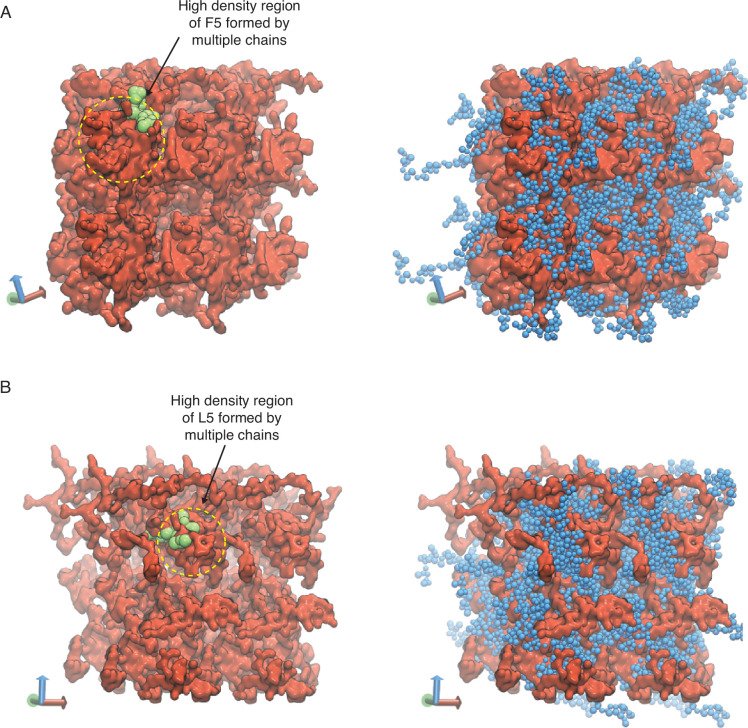
Representative configurations of (**A**) V_5_F_5_ and (**B**) V_5_L_5_ condensates from MARTINI simulations. The valine substituted half of the chain is colored blue (V_5_) and the X-substituted half of the chain is colored red (X_5_). To highlight the interpenetrating networks formed by the two halves, only the X-substituted half of the chain is shown on the left. Simulation interfaces are once repeated periodically in the positive x and positive y dimensions for clarity. High-density regions formed by the multiple X-substituted half of the chains are highlighted in yellow circles, with one of the chain shown in green.

**Figure 2—figure supplement 5.**
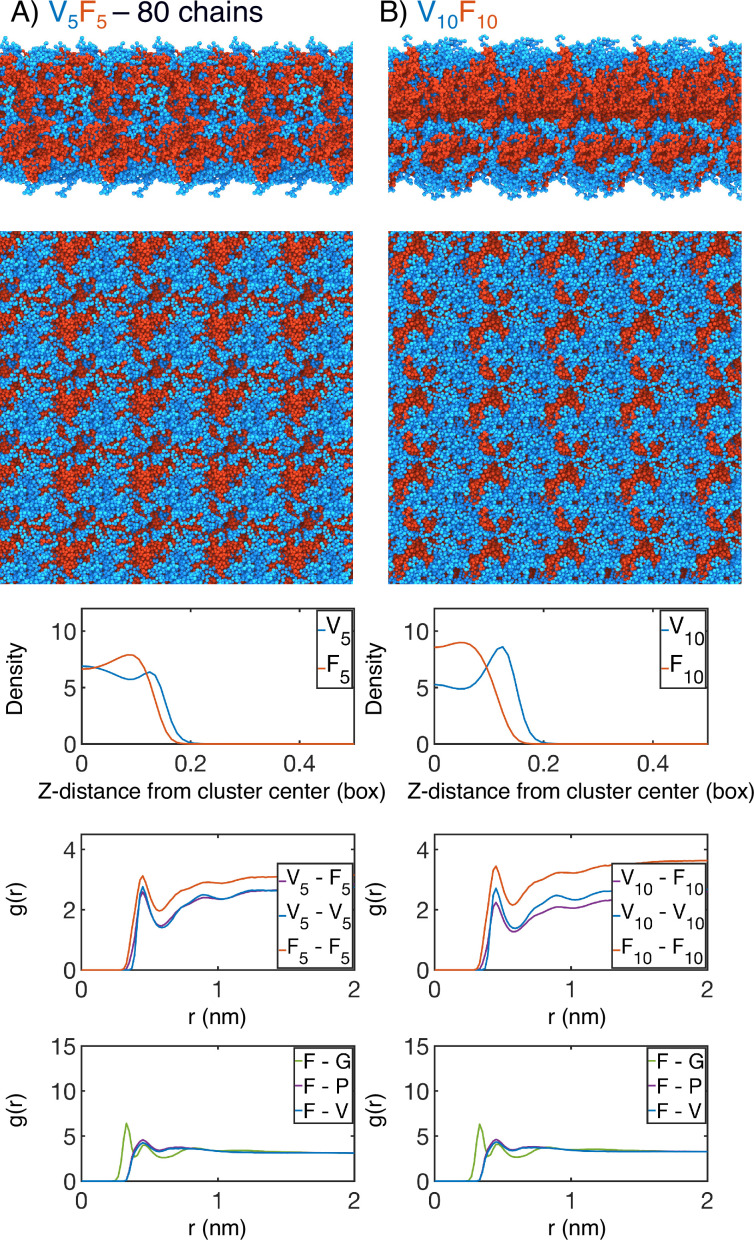
Finite size effect checks of our simulation methodology by (**A**) doubling the number of chains in the simulation box (V_5_F_5_ ; 80 chains) or by (**B**) doubling the length of the peptide sequence (V_10_F_10_). In each case, the uppermost panel is a periodic image of the simulated condensate. Only the protein is shown, with red highlighting the X-substituted half of the condensate and blue highlighting the V-substituted half of the condensate. The second panel from the top is condensate viewed from the simulation interface. The middle panel is the mass density from the condensate center for the V-substituted and X-substituted halves of each condensate. The second panel from the bottom is the radial distribution function g(r) for inter-chain coarse-grained beads, divided between the V_5_ half of the chain and X_5_ half of the chain. The bottom panel is the overall radial distribution function from the guest amino acid (X_5_), to those amino acids native to the ELP sequence.

**Figure 2—figure supplement 6.**
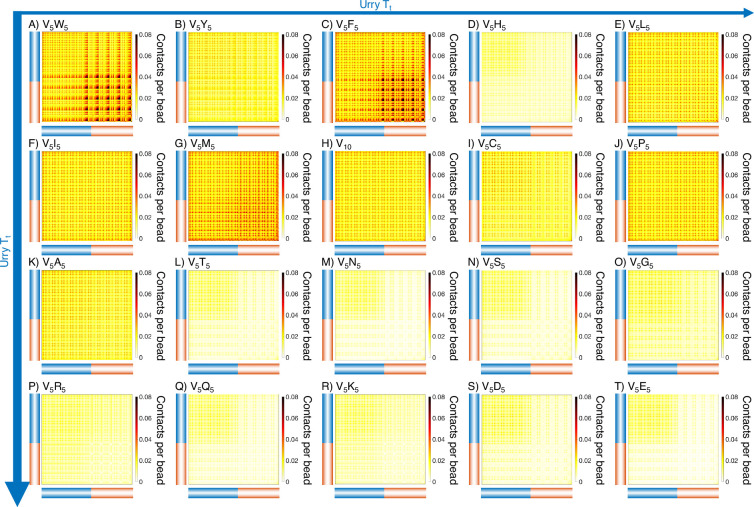
Intermolecular contact maps for elastin-like polypeptide (ELP) peptides from MARTINI simulations. The blue bar highlights the V_5_ half of the condensate, while red bar highlights the X_5_ half. Proteins (**A-T**) are arranged by increasing $T_{t}$ (decreasing hydrophobicity) from Urry, 1997 with (**A**, V_5_W_5_) being the most hydrophobic and (**T**, V_5_E_5_) being the least hydrophobic.

**Figure 2—figure supplement 7.**
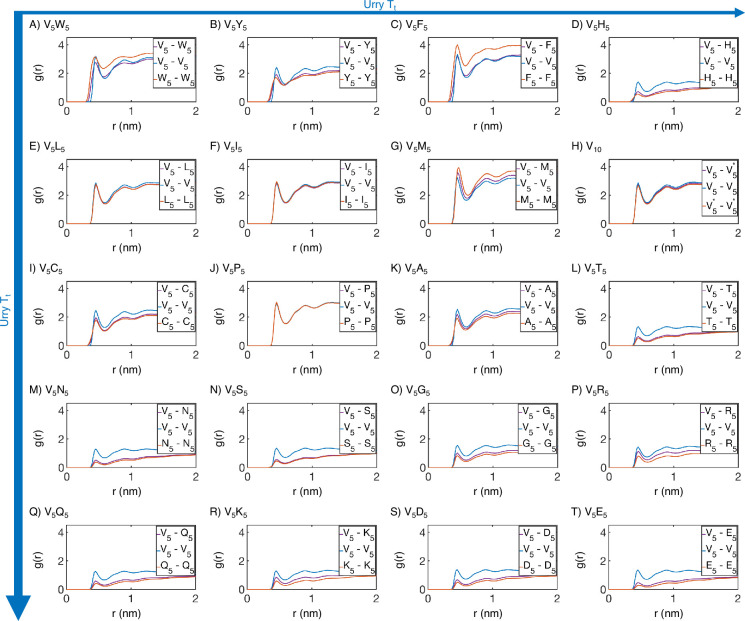
Radial distribution functions g(r) from MARTINI simulations support the microphase separation of different elastin-like polypeptide (ELP) blocks. Amino acids from the V_5_ and X_5_ half of the ELP peptide are partitioned into two blocks, and we used distances within and across the blocks to compute g(r). Distances between amino acids from the same peptide are excluded from computations. Proteins (**A-T**) are arranged by increasing $T_{t}$ (decreasing hydrophobicity) from Urry, 1997, with (**A**, V_5_W_5_) being the most hydrophobic and (**T**, V_5_E_5_) being the least hydrophobic.

Surprisingly, microphase separation did not produce lamellar morphology as expected for block copolymers with equal volume fraction of the two blocks (Figure 2—figure supplement 3; Bates and Fredrickson, 1999; Mai and Eisenberg, 2012; Zhulina et al., 2005; Leibler, 1980; Bates and Fredrickson, 1990; Matsen and Schick, 1994; Matsen and Bates, 1996; Grason, 2006; Swann and Topham, 2010; Shi, 2021). In particular, the condensates appear to form gyroid-like structures, in which the V and X blocks form two interpenetrating networks (Figure 2—figure supplement 4). This morphology also differs from micelle-like structures seen in simplified hydrophobic-polar (HP) polymers (Statt et al., 2020; Wessén et al., 2022). It promotes interfacial contacts while maintaining substantial self-interactions as well. Weak interfacial tension between different ELP blocks has also been noted by Hassouneh et al., 2015. These qualitative observations are insensitive to the polymer length and system size in our simulations (Figure 2—figure supplement 5). While longer diblock copolymers drive more prominent microphase separation, similar gyroid structures can also be observed. Notably, for more hydrophilic X blocks, the condensates begin to dissolve into a collection of micelle-like structures, consistent with the predictions by Hassouneh et al. in dilute solutions (Hassouneh et al., 2015).

Quantitative characterization of the condensate interior supports the presence of interpenetrating networks with substantial interfacial contacts as well. For example, significant contacts between V and X blocks can be readily seen in the inter-chain contact maps (Figure 2, Figure 2—figure supplement 6). We further computed the radial distribution functions for amino acids in various blocks (Figure 2, Figure 2—figure supplement 7). For all systems, we found that the probability of finding cross-block contacts V-X is often comparable to the intra-block contacts between X-X or V-V, depending on the relative hydrophobicity of V and X. For example, the more hydrophobic F blocks exhibit strong self-clustering, much more prominent than V-V blocks that are comparable to V-F blocks. On the other hand, clustering among V-V blocks and V-A blocks is more substantial than among A-A blocks. These results are again robust with respect to system setups (Figure 2—figure supplement 5).

**Figure 3.**
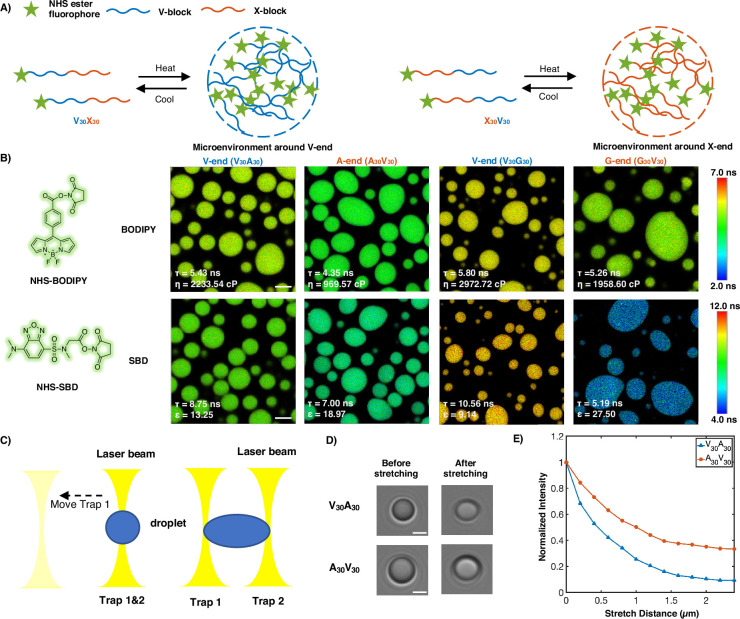
Experimental support of the microphase separation of elastin-like polypeptide (ELP) condensates. (**A**) Reversible ELP condensates formation via changing temperature. NHS ester fluorophores are attached at the amino-termini of V_30_X_30_ and X_30_V_30_ to detect the different micro-physicochemical properties between the V-end and the X-end. (**B**) Structures of NHS-BODIPY and NHS-SBD, and FLIM images of V_30_A_30_, A_30_V_30_, V_30_G_30_, and G_30_V_30_ labeled with respective fluorophores. The fluorescence lifetime of each image is the average acquired from three independent experiments. Scale bar: 5 μm. (**C**) Schematic diagram of optical tweezers stretching experiment. (**D**) Bright-field images of V_30_A_30_ and A_30_V_30_ in the stretching experiment using the optical tweezer. Scale bar: 2 μm. (**E**) Normalized droplets fluorescence intensity changes while stretching (red curve: V_30_A_30_; blue curve: A_30_V_30_). Fifteen droplets (at size ∼4 μm) were imaged and used for statistical analysis.

To experimentally test the microphase separation behavior uncovered in simulations, we studied the micro-physicochemical properties of the V-end and X-end of the peptides. We constructed diblock peptides with the combination of 30 pentameric repeats of V block and X (A or G) block, namely V_30_A_30_ and V_30_G_30_ (‘Experimental sequences’ in Appendix 1). The amino-termini of V_30_A_30_ and V_30_G_30_ sequences were subsequently labeled with environmentally sensitive BODIPY or SBD fluorophores (Liu et al., 2017; Shen et al., 2023), whose lifetime could be measured to quantify the viscosity or polarity of the V-end (Figure 3A, left panel) (Chambers et al., 2018). These probes have been reported to be only sensitive to single physicochemical properties (Ye et al., 2024; Shen et al., 2023). To avoid artifacts induced by fluorophore labeling, we usually used ELPs labeled with a low fraction of dyes. We also constructed A_30_V_30_ and G_30_V_30_ diblock peptides, wherein the viscosity or polarity of the A-end or the G-end could be measured by fluorophores that are attached at the amino-terminus (Figure 3A, right panel).

**Figure 3—figure supplement 1.**
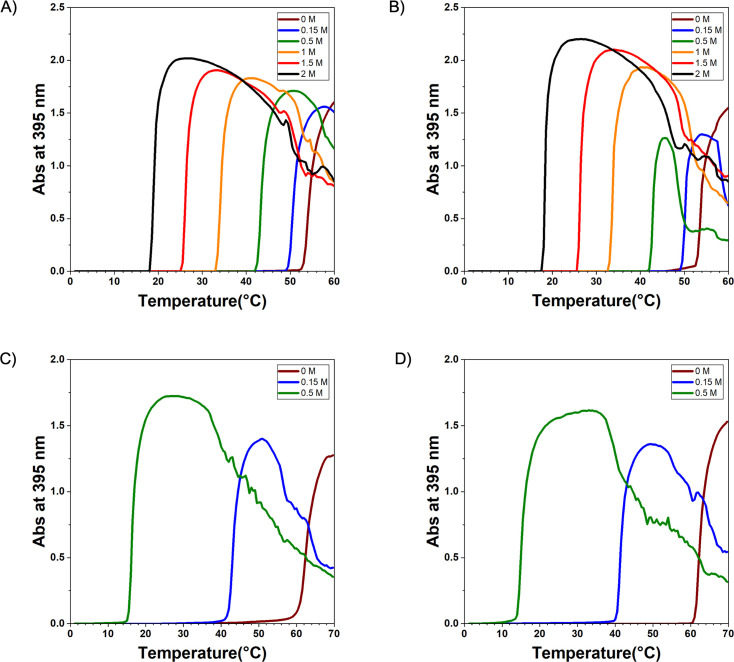
Experimental $T_{t}$ of elastin-like polypeptide (ELP) sequences. The $T_{t}$ diagram of (**A**) V_30_A_30_, (**B**) A_30_V_30_, (**C**) V_30_G_30_, and (**D**) G_30_V_30_. The turbidity was measured in the NaCl solution with different concentrations for V_30_A_30_ and A_30_V_30_ and in (NH_4_)2SO_4_ solution with different concentrations for V_30_G_30_ and G_30_V_30_. The heat-up rate is 0.5°C/min, and absorbance data were collected every 0.5°C.

**Figure 3—figure supplement 2.**
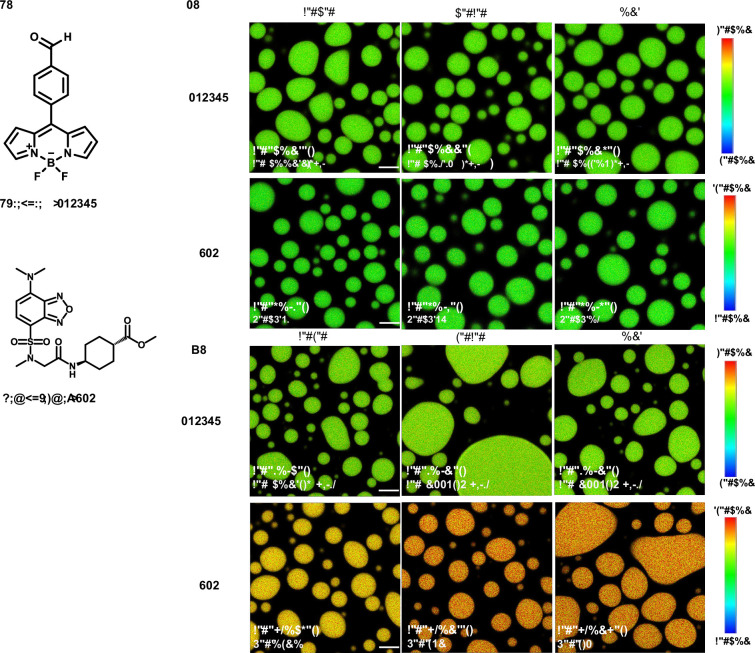
Fluorescence lifetime imaging microscopy (FLIM) image of unlabeled elastin-like polypeptide (ELP) condensates. (**A**) Chemical structure of free fluorophore, which can measure the physicochemical properties of condensates without labeling. (**B**) Representative FLIM images of V30A30 and A30V30. The mix is the mixture of V30A30 (35 μM) and A30V30 (35 μM). Droplets were formed with a final concentration of 70 μM ELP in 2 M NaCl solution with 1 μM fluorophore. (**C**) Representative FLIM images of V30G30 and G30V30. Droplets were formed with a final concentration of 70 μM ELP in 1.5 M (NH_4_)2SO_4_ solution with 1 μM fluorophore. The mix is the mixture of V30G30 (35 μM) and G30V30 (35 μM). Scale bar, 5 μm. The fluorescence lifetime of each image is the average from three independent measurements.

Using FLIM, we found that the lifetime of BODIPY for the V-end (5.43 ns) was longer than that for the A-end (4.35 ns), suggesting that the V-end indeed has a higher microviscosity than the A-end (*η_V_* = 2233.54 cp vs *η_A_* = 969.57 cp). Accordingly, the lifetime of SBD was longer for the V-end (8.75 ns) than the A-end (7.00 ns), indicating that the micropolarity of the V-end was lower than the A-end (ϵ_V_=13.25 vs ϵ_A_ = 18.97). These observations could be largely attributed to the greater extent of dehydration at the V-end due to its higher local peptide density. We further showed that the observed differences are not results of possible artifacts arising from any subtle distinctions between the two sequences V_30_A_30_ and A_30_V_30_ (‘Experimental characterization of ELP condensates’ in Appendix 1, Figure 3—figure supplements 1 and 2). Similar results were observed using the V-G sequences. FLIM experiments revealed that the V-end was more viscous than the G-end (*η_V_*=2972.72 cp vs *η_G_* = 1958.60 cp) and the V-end was less polar than the G-end (ϵ_V_=9.14 vs ϵ_G_ = 27.50). These experimental observations provided the first line of evidence to support the microphase separation, as suggested by the simulation results. It is worth noting that the reported values, although related, may not quantitatively represent the steady-state viscosity. This discrepancy arises from the slow relaxation timescale inherent in ELP condensates with viscoelastic properties.

To further investigate these phenomena in real time in motion, optical tweezers were used to stretch the droplet to observe the viscosity changes of different blocks. A single diblock condensate was captured by two optical traps (traps 1 and 2) at the same position, and it was stretched as trap 1 moved away from the original position (Figure 3C and D). We used the peptides V_30_A_30_ and A_30_V_30_, with BODIPY labeled at their amino-terminus, to study the microviscosity changes where the fluorescence intensity decreases as the viscosity decrease. We found that the normalized fluorescence intensity of the V-end decreases greater than the A-end, indicating that the viscosity change of the V-end is larger (Figure 3E). We anticipate that during stretching condensates deform and molecules may deviate from the preferred chemical environment established with microphase separation. Such deviations impact the hydrophobic segments more, leading to more significant reduction in their interactions with surrounding molecules and correspondingly their viscosity.

### Frustration produces condensate interior with interfacial properties

**Figure 4.**
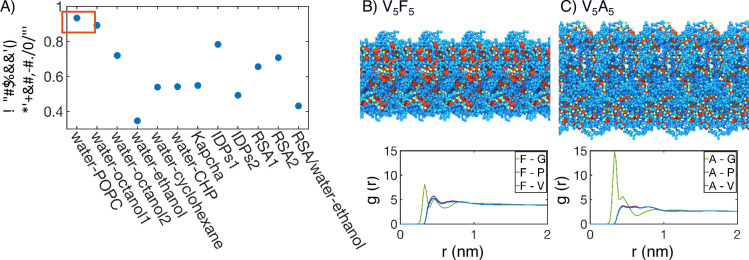
Interior of elastin-like polypeptide (ELP) condensates exhibits interfacial properties as a result of microphase separation. (**A**) Pearson correlation coefficient, ρ, between the stated measure of hydrophobicity and the condensate transition temperature, $T_{t}$, measured by Urry, 1997. To remove discrepancies in which end of the scale is hydrophobic and which end is hydrophilic, all hydrophobicity scales, including the Urry scale, are first normalized such that 1 corresponds to the most hydrophobic residue and 0 corresponds to the least hydrophobic residue. Hydrophobicity measures considered include experimental measures of water-solvent transfer free energies water-1-palmitoyl-2-oleoyl-sn-glycero-3-phosphocholine [POPC]-interface (White, 1996), water-octanol1 (Wimley et al., 1996), water-octanol2 (Wilce et al., 1995), water-ethanol (Zimmerman et al., 1968; Wilce et al., 1995), water-cyclohexane (Radzicka and Wolfenden, 1988), and water-N-cyclohexyl-2-pyrrolidone [CHP] (Lawson et al., 1984), atomic level analysis of moieties within amino acids (Kapcha and Rossky, 2014), computational approaches to estimate hydrophobicity within disordered proteins (IDPs1 Tesei et al., 2021 and IDPs2 Dannenhoffer-Lafage and Best, 2021), bioinformatics techniques that approximate the burial propensity, or relative solvent accessibility (RSA) of amino acids (RSA1 Rose et al., 1985 and RSA2 Tien et al., 2013), and a method that mixed protein burial fraction with water-vapor transfer free energy (RSA/water-vapor Kyte and Doolittle, 1982). The red box highlights the high correlation between $T_{t}$ and water-octanol or water-POPC interface transfer free energies. (**B, C**) Condensate organization from MARTINI simulations for V_5_F_5_ (**B**) and V_5_A_5_ (**C**). The top panels provide protein-only views for simulated condensates, with the guest residue X, glycine adjacent to the guest residue, and the remaining residues colored red, green, and blue, respectively. Condensate images are repeated periodically in the x/y plane. The bottom panel shows the overall radial distribution function from the guest amino acid to those amino acids native to the ELP sequence.

The strong dependence of molecular organization on amino acid hydrophobicity suggests that the solvation environment of individual residues might be a determining factor for condensate stability. Indeed, as shown in the ‘Supplemental theory’ of Appendix 1, the critical temperature is closely related to the free energy cost of transferring polymer beads from a solution state to a polymer-only environment. This transfer free energy is often used to quantify the hydrophobicity of amino acids (Regy et al., 2021; White, 1996; Wimley et al., 1996; Wilce et al., 1995; Zimmerman et al., 1968; Radzicka and Wolfenden, 1988; Lawson et al., 1984). To explore their relationship more quantitatively, we compared the transition temperature for ELP condensates measured by Urry, 1997 to several hydrophobicity scales. Figure 4A shows that water-octanol and water-POPC interface transfer free energies best correlate with the transition temperature. Meanwhile, other hydrophobicity measures, even including those derived specifically for disordered proteins, do not match as well. We note that our conclusion here is not dependent on the limited number of hydrophobicity measures examined in this study, and can be seen in a previous clustering of 98 different hydrophobicity scales (Simm et al., 2016).

**Figure 4—figure supplement 1.**
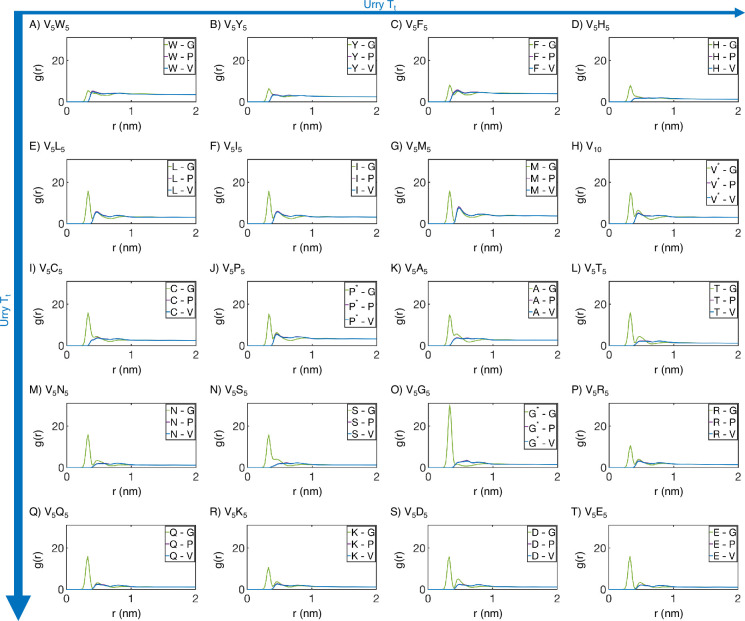
Residue-specific radial distribution function g(r) from MARTINI simulations. Distances from the guest amino acid (X) to those amino acids native to the elastin-like polypeptide (ELP) sequence were used to compute g(r). Substituted amino acids that also appear in the native sequence are differentiated from their native counterparts using a star (*). Proteins (**A-T**) are arranged by increasing $T_{t}$ (decreasing hydrophobicity) from Urry, 1997, with (**A**, V_5_W_5_) being the most hydrophobic and (**T**, V_5_E_5_) being the least hydrophobic.

The correlation analysis of the Urry transition temperature with various hydrophobicity scales supports a unique chemical environment emerging from the collective behaviors of condensates. To better understand the microenvironment surrounding guest residues in X blocks, we computed the radial distribution functions using MARTINI simulations. As shown in Figure 4B and C, Figure 4—figure supplement 1, the guest residues are immediately surrounded with hydrophilic glycine residues as the nearest neighbors. In the second shell, more hydrophobic valine and proline residues appear. These profiles reveal the interfacial nature of the chemical environment surrounding the guest residues consisting of both hydrophobic and hydrophilic amino acids. This interfacial solvation environment is consistent with the strong correlation of the Urry scale with water-POPC interface transfer free energy. Therefore, ELP condensates do not simply feature a hydrophobic interior that separates from hydrophilic residues as seen for folded proteins, and hence the poor correlation shown in Figure 4A with the burial propensity of amino acids (RSA1, RSA2).

The ELP peptide sequences partly dictate the interfacial environment. For example, the guest residues are juxtaposed between two glycine residues in the pentamer (Figure 1A), creating the immediate shell of hydrophilic residues. However, the presence of valine in the pentamer prevents the movement of GXG motifs to the interface due to the clustering of hydrophobic residues that prefer the condensate interior. Therefore, while microphase separation occurs in ELP condensates, frustration remains in the system. Hydrophilic residues cannot completely separate from hydrophobic ones due to constraints imposed by the amino acid sequence, creating unique microenvironments. Such a microenvironment arises from the collective behavior of many proteins and can deviate from individual chains (Wei et al., 2017), explaining the less-than-ideal correlation between the Urry scale and the hydrophobicity scales optimized to reproduce the conformational ensemble of IDP monomers.

### Solvation environment from atomistic simulations support interfacial properties

The interfacial environment uncovered in the previous section suggests the presence of significant solvation in the condensate interior. For condensates with lower surface tension, such as V_5_G_5_, the protein density near the center of the condensate is only around ∼30%. Even for the stable condensates with high surface tension, such as V_5_F_5_, the protein density near the center only reaches ∼70%. The relative density of protein, water, and ions in different systems generally follows the trend observed for the transition temperature (Appendix 1—figures 1 and 2).

**Figure 5.**
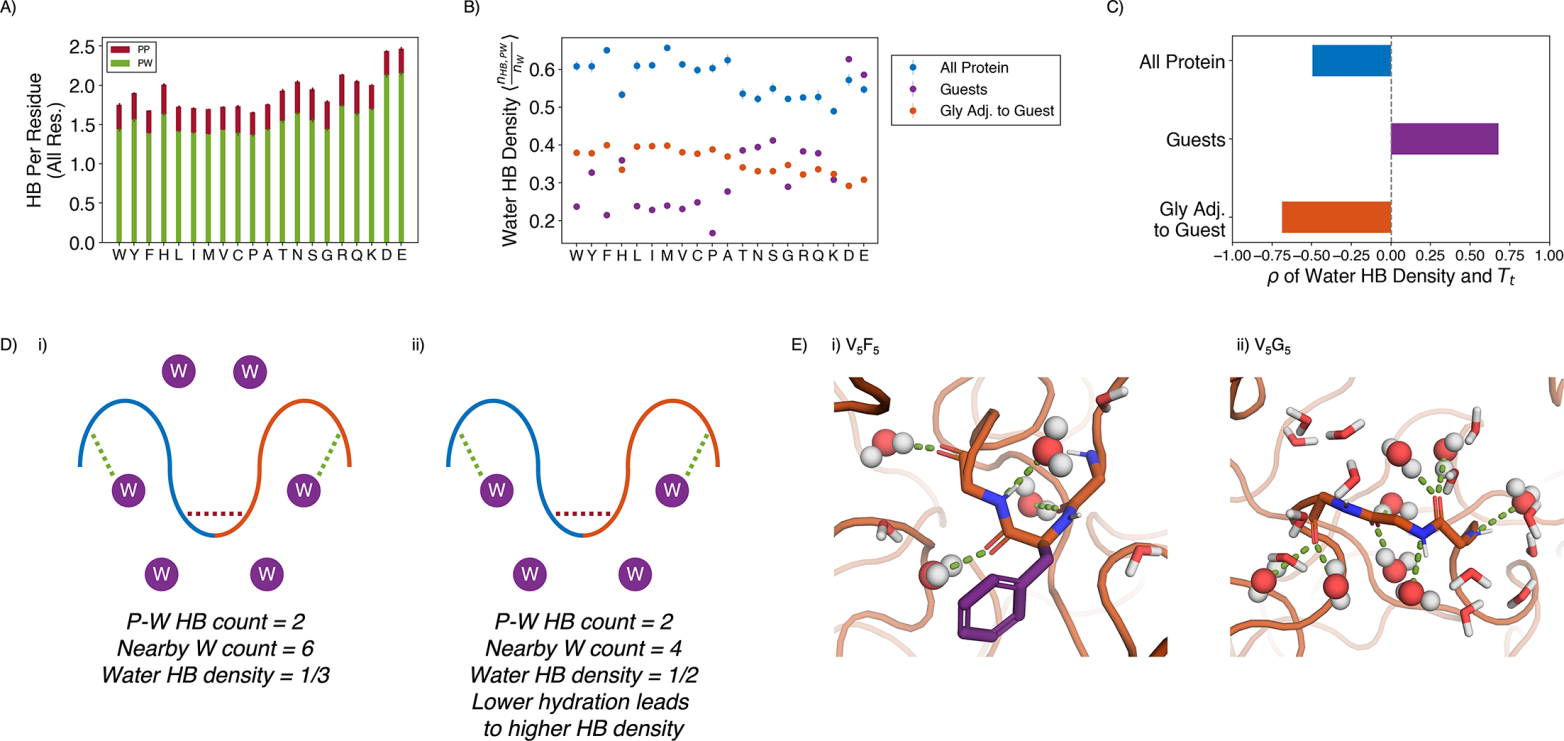
Water hydrogen bonding environment of condensates from all-atom simulations. (**A**) Bar chart depicting the average number of protein-water (PW, in green) and protein-protein (PP, in red) hydrogen bond (HB) per residue for each condensate system. The x-axis presents the guest residue of the system and is ordered by the Urry scale. Error bars represent SDs of four independent estimates. (**B**) Water HB density for each of the 20 condensates, for three different residue selections. The metric is shown for all residues in the system (blue), guest residues (purple), and glycine residues adjacent to the guest residue in the sequence (orange). The x-axis presents the guest residue of the system and is ordered by the Urry scale. Error bars represent SDs of four independent estimates. (**C**) Correlation coefficients of the water HB density shown in (B) with Urry $T_{t}$. (**D**) Schematics depicting two systems with low (i) and high (ii) water hydrogen bond density. The protein is depicted as blue and orange lines, and the water molecules are depicted as purple circles. Protein-protein and protein-water hydrogen bonds are drawn as red and green dashed lines respectively. (**E**) Visualizing hydrogen bond density with illustrative snapshots for V_5_F_5_ (i) and V_5_G_5_ (ii) condensate. Side chain carbon atoms are shown in purple. Water molecules near the protein are explicitly depicted, with oxygen and hydrogen atoms colored in red and white. Water molecules hydrogen bonded to the protein are depicted as spheres (with hydrogen bonds shown in green lines).

The large water mass fractions in the condensates prompt the question of the extent to which water is engaged in meeting the protein’s hydrogen bonding requirements.We determined the average number of hydrogen bonds per residue with ELP residues or water from extensive atomistic simulations. As shown in Figure 5A, over 75% of the hydrogen bonds are between protein and water in all of the condensates. Prior work on a different ELP condensate (Rauscher and Pomès, 2017) similarly indicated that water-protein hydrogen bonds constitute most of the hydrogen bonds found in the system. This finding highlights that ELPs, lacking noticeable secondary structures (Figure 5—figure supplement 1), are less capable of meeting its own hydrogen bonding needs, compared to folded proteins (Sticke et al., 1992). Therefore, high water concentration within the condensate is crucial to fulfilling the protein’s hydrogen bonding requirements.

**Figure 5—figure supplement 1.**
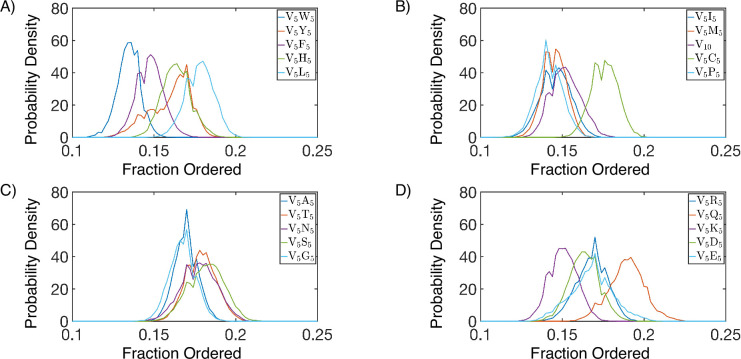
Secondary structure of simulated protein condensates, splitted into four panels (**A-D**), with protein names indicated in the legends. Secondary structure is defined according to the DSSP algorithm (Touw et al., 2015; Kabsch and Sander, 1983). We consider α-helices, β-sheets, β-bridges, and turns as ordered, and display the total fraction of all these secondary structure elements for each simulated condensate.

Strikingly, given the importance of water molecules, the average number of hydrogen bonds per residue remained relatively constant across condensates, even when the amount of water inside the condensate varied by almost two folds. Therefore, we hypothesize that each water molecule in more hydrophobic condensates must engage more in hydrogen bonding than each water in hydrophilic condensates. To test this hypothesis, we determined the water hydrogen bond density, defined as the ratio of the number of protein-water hydrogen bonds divided by the total number of water molecules near the condensate (Figure 5D and E). As shown in Figure 5B and C, the water hydrogen bond density indeed negatively correlates with the condensate transition temperature, $T_{t}$, supporting our hypothesis that more hydrophobic condensates would have a higher hydrogen bond density.

The water hydrogen bond density also highlights an interfacial environment of blended hydrophobic and hydrophilic regions. For example, when we limit the analysis to water molecules near the guest residues, X, the hydrogen bond density now becomes positively correlated with $T_{t}$. Therefore, locally, protein-water interactions are strongly influenced by the chemistry of side chains, and water molecules have fewer hydrogen bonding opportunities with more hydrophobic residues. However, the opposite trend is observed for the hydrogen bond density near glycine residues that immediately surround the guest residues. Therefore, the exposed backbones retain water molecules even when hydrophobic side chains do not provide hydrogen bonding opportunities, and the inseparability between the two further contributes to the observed interfacial property of condensates.

**Figure 6.**
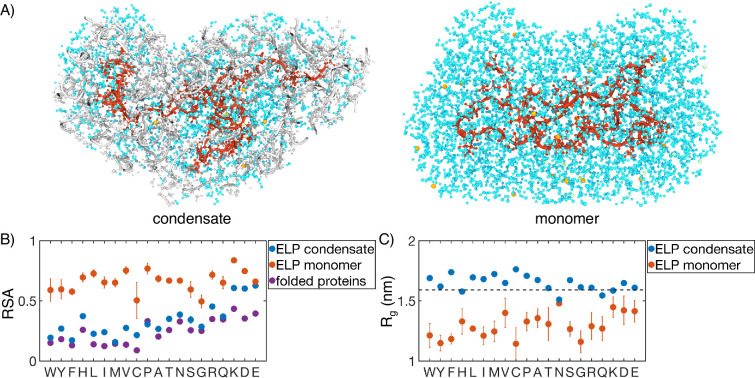
Comparison of protein conformation and solvation between elastin-like polypeptide (ELP) condensates and monomers. (**A**) Zoom-in view of a single peptide chain (red) from atomistic simulations of V_5_A_5_ condensate (left) and monomer (right). Atoms within 1 nm of the peptide are shown, including water (cyan), chlorine (green), sodium (orange), and protein atoms from other chains (gray). (**B**) Relative solvent accessibility (RSA) of guest residues estimated from atomistic simulations of condensates (blue) and monomers (orange). For comparison, the corresponding values estimated using folded proteins are included as purple dots (Tien et al., 2013). Error bars represent the SD of four independent time estimates. (**C**) Comparison of the radius of gyration ($R_{g}$) for peptides estimated from atomistic simulations of condensates (blue) and monomers (orange). The dashed line represents the expected $R_{g}$ of an ideal chain, utilizing values that have been previously suggested for intrinsically disordered proteins (IDPs) (Hofmann et al., 2012; Dignon et al., 2018a). Error bars represent the SD of four independent time estimates.

As a complementary measure of the solvation environment of individual residues, we computed the relative solvent accessibility (RSA) for the guest amino acids using atomistic simulations (Figure 6A and B). The RSA measures the solvent-accessible surface area (SASA) normalized by the maximum possible solvent exposure. RSA values in ELP condensates are much lower than those computed from all-atom simulations of ELP monomers, consistent with the increase of polymer density upon phase separation. However, they are almost always higher than the values estimated for folded proteins due to many water molecules roaming inside the condensates (Tien et al., 2013). Therefore, while ELP condensates provide a mechanism to shield hydrophobic amino acids from solvent, they are less effective in doing so than folded proteins.

**Figure 6—figure supplement 1.**
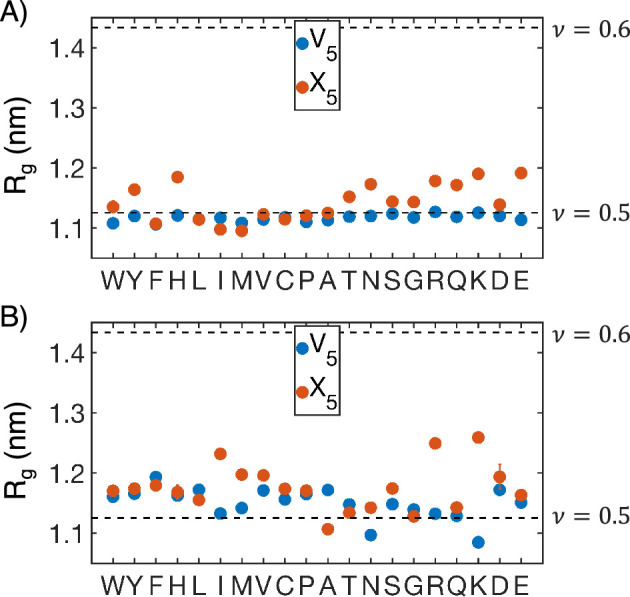
$R_{g}$ of individual peptide blocks from MARTINI (**A**) and atomistic (**B**) simulations. V_5_ represents the valine block, while X_5_ represents the guest block. Error bars represent SDs of the mean over four independent time windows. The dashed lines represent different values of polymer scaling exponents, with ν=0.5 corresponding to an ideal chain and ν=0.6 corresponding to a swollen polymer chain. Values previously suggested for intrinsically disordered proteins (IDPs) were used in calculation of the scaling exponent (Hofmann et al., 2012).

While the amino acid RSA values increase upon phase separation, unlike protein folding, the increase does not result from polymer collapse. We observe a significant expansion for almost all the studied peptides upon condensation, supporting the notion that water is a poor solvent for ELP (Figure 6C). The expansion is most evident for more hydrophobic ELPs, the radii of gyration of which are now comparable to that of an ideal Gaussian chain. Notably, the sizes of the two separate peptide blocks are similar in most systems, supporting an interfacial environment that can accommodate both hydrophobic and hydrophilic residues (Figure 6—figure supplement 1).

## Discussion

We carried out multiscale simulations to elucidate the connection between protein sequences and emergent properties of ELP condensates. The multiscale approach overcomes challenges in sampling slow conformational rearrangements to produce equilibrium atomistic condensate configurations. The simulations successfully reproduced condensate stability variation upon amino acid substitution. While our study is performed at set salt concentration and temperature to isolate the contributions of amino acid hydrophobicity to condensate organization, future studies may consider implementing temperature (Dignon et al., 2019) or salt (Wohl et al., 2021)-dependent models to explore how solution conditions affect the organization of ELP condensates.

We found that diblock ELPs undergo microphase separation, with the more hydrophobic blocks localizing toward the condensate interior. The more hydrophobic blocks increase the local protein density, resulting in higher viscosity and lower dielectric constants, as determined in FLIM experiments. In contrast to the interior of folded proteins, these blocks are significantly solvated with water occupying over 30% of mass density. Unlike the weak micelles formed in solution (Hassouneh et al., 2015), ELP condensates exhibit gyroid-like morphologies that promotes contacts between blocks. Further studies on the thermodynamic stability of these morphologies and comparing with predictions from the self-consistent field theory shall provide more insights into the driving forces for their emergence.

Our study uncovered a remarkably simple grammar for determining the stability of ELP condensates. A single quantity specific to the guest residue suffices to predict the emergent properties of the condensate, as evidenced by the strong correlation of the transition temperatures with the transfer free energies. The lack of higher-order effects is striking, supporting a mean field approximation to account for the contribution of individual residues in the system. However, the hydrophobicity scale is itself sensitive to the local microenvironment that is sequence specific. Such a microenvironment arises from the collective behavior of many proteins, can deviate from that of individual chains, and is likely sensitive to the solution conditions (Welsh et al., 2022), which are held constant in our study. Future work on systems with double amino acid substitutions or changes to salt concentration or temperature could elucidate the generality of the mean field interpretation and the additivity of individual contributions.

While the transferability of the extracted rule for condensate stability to other systems remains to be shown, there are reasons to be hopeful. For instance, we anticipate that the microphase separation that produces the interfacial properties is a general principle for condensate organization. For most IDPs, the hydrophobic and hydrophilic residues are often interspersed throughout the chain to prevent the collapse of individual proteins (Martin et al., 2020; Zheng et al., 2020) and, correspondingly, the complete phase separation as in the lamellar phase. In addition, the hydrophilic backbones are expected to be exposed due to a lack of secondary structure formation, contributing to the interfacial property in the presence of hydrophobic side chains. As such, we anticipate that future research will discover the broad presence of interfacial environments within microphase separated motifs, as observed here.

## Methods

### Molecular dynamics simulation details

To better understand the properties of biological condensates, we performed simulations on 40 diblock ELPs with the sequence (V-P-G-V-G)*_n_*-(V-P-G-X-G)*_n_*. We set n=5 to balance computational efficiency and polymer topological effects. As shown in Figure 2—figure supplement 5, the results presented in the main text are qualitatively robust with respect to the system size and peptide length.

The multiscale simulations of ELP condensates started with an implicit solvent model, MOFF (Latham and Zhang, 2021), and a similar homopolymer model, to generate initial configurations for each system. Next, we converted the α-carbon-only configurations into those consistent with the MARTINI force field (Souza et al., 2021) for coarse-grained, explicit solvent simulations. These simulations measured the coexistence between the condensate and solvent, lasted for 100 μs, and were conducted in the NP_N_AT ensemble. Finally, we converted the MARTINI configurations into atomistic structures for simulations with CHARMM36m (Huang et al., 2017) force field, with protein-water interactions shifted to better characterize IDPs. Atomistic simulations were conducted in the NP_N_AT ensemble and lasted 250 ns. All simulations were conducted using the software GROMACS (Berendsen et al., 1995) and are described in detail in ‘Condensate simulation details’ in Appendix 1.

In addition to the condensates, we performed atomistic simulations of ELP monomers. These simulations began from RoseTTAFold predictions of the peptide structure (Baek et al., 2021; Bonneau et al., 2001) and lasted for 1 μs in the NPT ensemble. Full details are available in ‘Monomer simulation details’ in Appendix 1.

### Analysis of condensate simulations

#### Surface tension

In explicit solvent simulations, two coexisting phases are present, in which proteins form a dense slab in the x-y plane, and the solvent creates a dilute phase in the z-dimension (Dignon et al., 2018b; Ladd and Woodcock, 1977). We controlled the pressure normal to the protein-solvent interface by performing simulations in the NP_N_AT ensemble (Zhang et al., 1995). The surface tension (τ) was calculated from these simulations as

(1) $$\tau =\frac{L_{z}}{2N}*(P_{ZZ}-\frac{1}{2}(P_{XX}+P_{YY})),$$

where $L_{z}$ is the length of the Z-dimension and N is the number of protein droplets. $P_{XX}$, $P_{YY}$, and $P_{ZZ}$ are the components of the pressure tensor along the three axes.

#### Clustering

Since the condensate can diffuse along the z-axis, that is, the direction perpendicular to the interface, we aligned the simulated configurations using the position of the largest cluster of peptide molecules. To find the largest cluster, we first computed a center of mass contact matrix between peptide molecules with a distance cutoff of 4 nm. Then, using a depth first search algorithm (Tribello et al., 2017), we identified the size and location of the largest protein cluster. The system was then shifted such that the z coordinate of the center of mass of the largest cluster is at zero. We implemented the alignment using the software MDAnalysis (Gowers et al., 2016; Michaud-Agrawal et al., 2011).

#### Contact map

Protein contact maps were calculated using a cutoff distance of 0.6 nm. Specifically, two residues were defined as in contact if any pair of their coarse-grained beads were within 0.6 nm.

#### Radial distribution function

Radial distribution functions were calculated using the InterRDF function of MDAnalysis between 0.00001 nm and 2 nm. For analyzing the microphase separation of each condensate, we divided each protein into the V-substituted blocks and X-substituted blocks. This radial distribution function excluded intra-chain contacts to better capture how inter-chain contacts determine condensate organization. To analyze the origin of chain frustration within diblock phase separation, we computed the radial distribution function of each guest amino acid to the amino acids native to the sequence. Intra-chain contacts were included to fully capture the local environment experienced by the guest amino acid.

#### Relative solvent accessibility

The relative solvent accessibility (RSA) was measured based on the SASA of guest amino acids determined from all-atom simulations. We first calculated the SASA for each residue using GROMACS tools sasa, with a probe radius of 0.14 nm (Eisenhaber et al., 1995). We then averaged across all repeats of guest amino acids and normalized the SASA by the maximum possible value for a given amino acid to compute RSA (Tien et al., 2013; see Appendix 1—table 1).

#### Radius of gyration

The estimated $R_{g}$ from atomistic simulations were compared to the following analytical expression (Hofmann et al., 2012):

(2) $$R_{g}=\sqrt{\frac{2l_{p}b}{\left(2\nu +1)(2\nu +2\right)}}N^{\nu },$$

where N is the length of the polymer, b=0.38 nm is the bond length, and $l_{p}=0.40$ nm is the persistence length.

#### Secondary structure

Secondary structure for protein condensates was calculated from atomistic simulations using the GROMACS do_dssp command (Touw et al., 2015; Kabsch and Sander, 1983). Figure 5—figure supplement 1 displays the ordered fraction of secondary structures, which includes α-helices, β-sheets, β-bridges, and turns.

#### Hydrogen bonding

Protein-protein and protein-water hydrogen bonds were determined using the hydrogenbonds package within MDAnalysis (Gowers et al., 2016; Michaud-Agrawal et al., 2011; Smith et al., 2019). A hydrogen bond was defined as having a donor heavy atom within 3.30 Å of an acceptor heavy atom and a donor heavy atom-hydrogen atom-acceptor heavy atom angle of 135.0° or larger. The nearby water count for each analysis selection was determined by counting the water molecules whose oxygen atoms were within 4.0 Å of the heavy atoms of this selection. Water hydrogen bond density for each residue selection for each frame was computed as the ratio of protein-water hydrogen bonds to the number of nearby water molecules (Appendix 1—figure 3).

### Experimental methods

#### Fluorescence lifetime imaging microscopy (FLIM)

The proteins of interest were labeled with NHS ester fluorophore. We used ELPs with 1% BODIPY labels or 2.5% SBD labels to form condensates, which avoid the artifacts induced by fluorophores. Droplets were formed with the final concentration of 70 μM ELP in 2 M NaCl for V-A and 1.5 M NH_4_SO_4_ for V-G diblock, respectively. A drop of droplets containing solution was placed on a 0.17 mm coverslip with a 500 μm spacer. Images were acquired using Leica Falcon Fluorescence Microscope equipped with Wil pulse laser and ×63/0.12 oil-immersion objective. The BODIPY was excited at 488 nm and the SBD was excited at 448 nm. The fluorescence lifetime fitting and image analysis were performed in LAS X and ImageJ.

### Optical tweezer experiments

The protocol describes the preparation and manipulation of a sample containing a BODIPY-labeled ELP protein using an optical tweezer, specifically the C-Trap from LULICKS. The goal is to form a single large condensate from small droplets and then stretch it using the optical tweezers while monitoring its fluorescence intensity. The first step is to mix the BODIPY-labeled ELP protein with NaCl solution to obtain a final concentration of 20 μM protein in 2 M NaCl. The sample is then loaded into the flow channel of the optical tweezer. The dual trap mode of the C-Trap is then used to move the two traps to the same position. The sample is flushed until a single large condensate of about 4 μm is caught by the dual trap via the fusion of small droplets. The traps are then moved along with the droplet to another flow channel containing 2 M NaCl buffer. The channel and pressure are turned off to avoid any additional force on the droplet. The next step is to stretch the droplet by moving trap 1 0.2 μm each time. Fluorescent images of each stretch are taken using a 488 nm excitation laser. The mean fluorescence intensity of the whole droplet is then quantified using ImageJ.

## Figures and Tables

**Appendix 1—figure 1.**
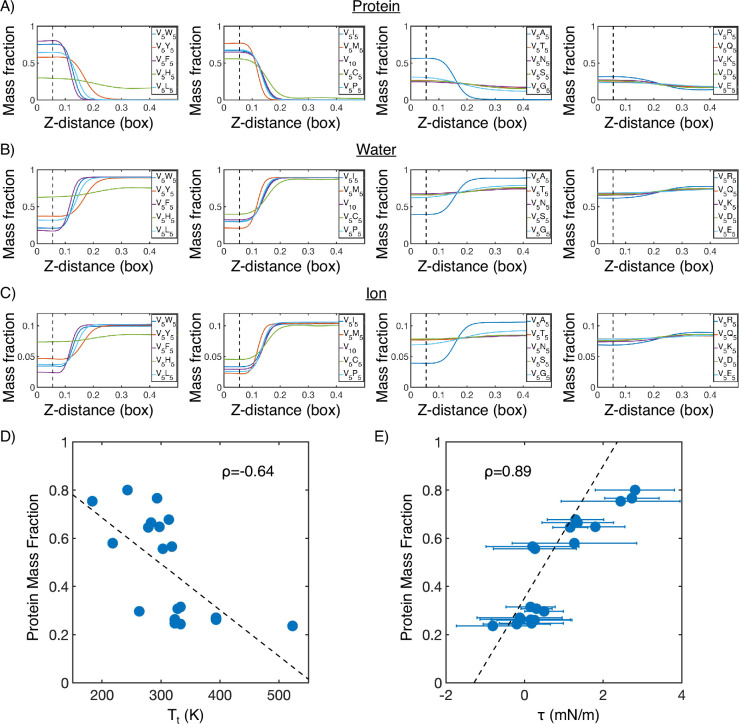
Mass fraction from MARTINI condensate simulations. (**A**) Protein mass fraction along the *Z*-distance from the condensate center defined using the largest cluster. The Z-axis is defined as the direction perpendicular to the condensate-water interface. The dashed line represents a *Z*-distance of 0.06 box lengths. Average values below this threshold are used for correlation analysis in (**D**) and (**E**). (**B**) Water mass fraction along the *Z*-distance from the condensate center. (**C**) Ion mass fraction along the *Z*-distance from the condensate center. (**D**) Correlation between the average protein mass fraction and transition temperature ($T_{t}$) from Urry, 1997. ρ is the Pearson correlation coefficient between the two data sets, and the dashed diagonal line is the best fit line. Error bars represent SDs of the mean and are smaller than the symbols. (**E**) Correlation between the average protein mass fraction and the simulated surface tension (τ). ρ is the Pearson correlation coefficient between the two data sets, and the dashed diagonal line is the best fit line. Error bars represent SDs of the mean taken over six equally spaced box length intervals.

**Appendix 1—figure 2.**
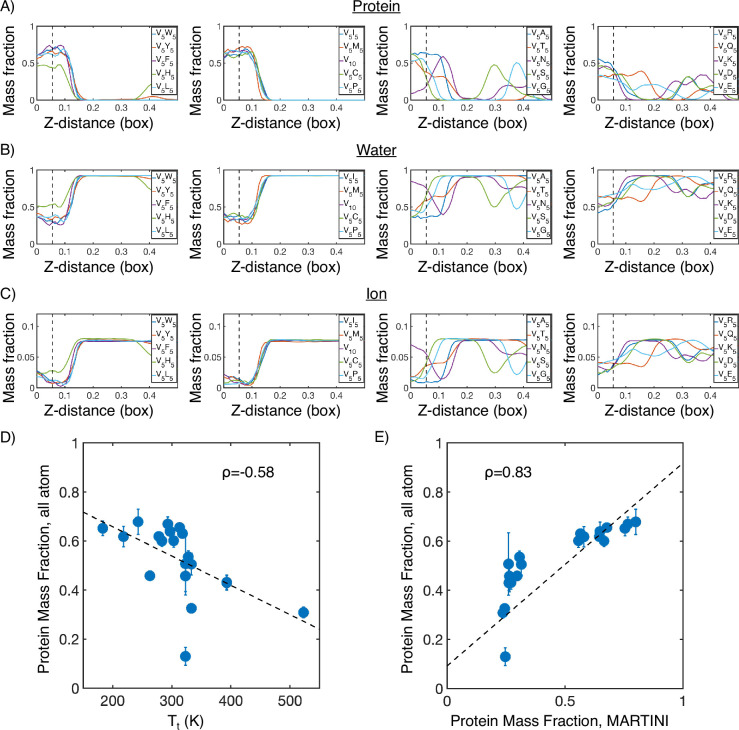
Mass fraction from atomistic condensate simulations. (**A**) Protein mass fraction along the *Z*-distance from the condensate center defined using the largest cluster. The Z-axis is defined as the direction perpendicular to the condensate-water interface. The dashed line represents a *Z*-distance of 0.06 box lengths. Average values below this threshold are used for correlation analysis in (**D**) and (**E**). (**B**) Water mass fraction along the *Z*-distance from the condensate center. (**C**) Ion mass fraction along the *Z*-distance from the condensate center. (**D**) Correlation between the average protein mass fraction in all-atom simulations and transition temperature ($T_{t}$) from Urry, 1997. ρ is the Pearson correlation coefficient between the two data sets, and the dashed diagonal line is the best fit line. Error bars represent SDs of the mean taken over six equally spaced box length intervals. (**E**) Correlation between the average protein mass fraction from all-atom simulations and the average protein mass fraction from MARTINI simulations. ρ is the Pearson correlation coefficient between the two data sets, and the dashed diagonal line is the best fit line. Error bars represent SDs of the mean taken over six equally spaced box length intervals.

**Appendix 1—figure 3.**
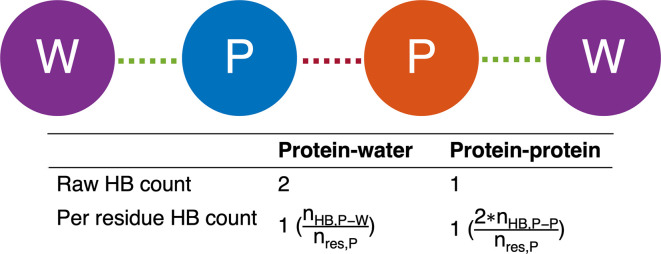
Graphical illustration of number of hydrogen bonds per protein residue. The protein is depicted as blue and orange circles, and the water molecules are depicted as purple circles. Protein-protein and protein-water hydrogen bonds are drawn as red and green dashed lines, respectively. The number of protein-water hydrogen bonds per protein residue is calculated by taking the raw number of protein-water hydrogen bonds ($n_{HB,P-W}$) and dividing by the number of residues in the protein ($n_{res,P}$). Meanwhile, the number of protein-protein hydrogen bonds per protein residue is calculated by doubling the number of protein-protein hydrogen bonds ($n_{HB,P-P}$) and dividing by the number of residues in the protein ($n_{res,P}$). The extra factor of 2 accounts for the fact that, in the case of protein-protein hydrogen bonds, both the donor and the acceptor must reside within the protein.

**Appendix 1—figure 4.**
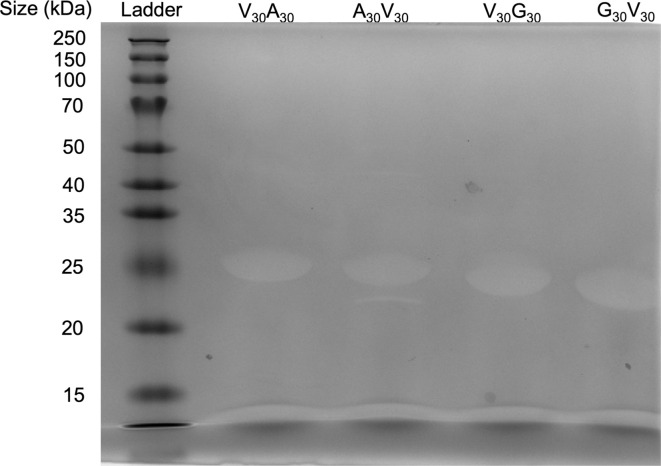
SDS-PAGE gel of elastin-like polypeptide (ELP). The purity of the protein is >95%. Appendix 1—figure 4—source data 1.Original files for western blot analysis displayed in Appendix 1—figure 4. Appendix 1—figure 4—source data 2.Original western blots for Appendix 1—figure 4, indicating the relevant bands and treatments.

**Appendix 1—figure 5.**
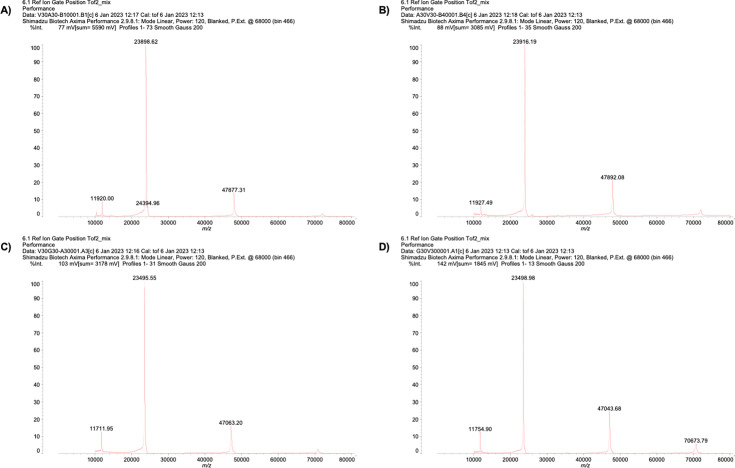
Mass spectrum of (**A**) V_30_A_30_, (**B**) A_30_V_30_, (**C**) V_30_G_30_, and (**D**) G_30_V_30_.

**Appendix 1—figure 6.**
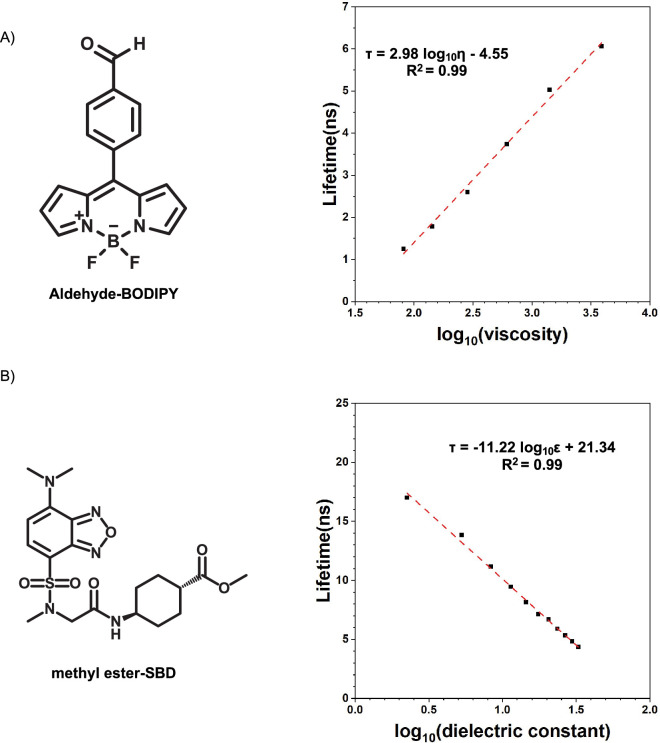
Calibration curves of fluorophores. (**A**) Lifetime-viscosity calibration curve is quantified in the glycerol at different temperatures (10°C *η* = 3900 cP; 20°C *η* = 1,412 cP; 30°C *η* = 612 cP; 40°C *η* = 284 cP; 50°C *η* = 142 cP; 60°C *η* = 81.3 cP) Segur and Oberstar, 1951?. The fluorophore concentration is 5 μM. (B) Lifetime-dielectric constant calibration curve is quantified in the MeOH-1,4-dioxane mixture. $\epsilon_{mix}=\phi_{1}\epsilon_{1}+\phi_{2}\epsilon_{2}=32.7$, $\epsilon_{1,4-dioxane}=2.25$.

## Data Availability

The current manuscript is a computational study, so no data have been generated for this manuscript.

## Additional files

MDAR checklist

## Appendix 1

### Condensate simulation details

We employed a multiscale approach to study ELP condensates. Our simulations consisted of four steps with increasing resolution. We start with homopolymer and MOFF (Latham and Zhang, 2021) simulations that help generate initial configurations for the peptides within the condensate. Then we use the results from the explicit solvent coarse-grained model, MARTINI (Souza et al., 2021), and all-atom explicit solvent force field, CHARMM36m (Huang et al., 2017), for further examination and quantitative analysis. Here, we describe the force field and simulation parameters at each step. Simulations were conducted using GROMACS (Berendsen et al., 1995). Homopolymer and MOFF configurations were performed on GROMACS 2018.4, which allowed for tabulated interactions not allowed in current versions of GROMACS. MARTINI and all-atom simulations were performed using GROMACS 2021.

### Homopolymer simulations

We used an identical homopolymer simulation to help generate starting configurations for elastin-like polypeptides. By using an identical configuration, all future simulations begin with the same molar density of protein, despite different sequences of ELPs. Our homopolymer force field was based on the MOFF force field. It assumed random coil secondary structure and non-bonded interactions identical to MOFF’s valine-valine interaction. More specifically,

(3) $$U_{hom}(r)=U_{bond}+U_{angle}+U_{contact},$$

where $U_{bond}$, $U_{angle}$, and $U_{contact}$ account for the bonds between neighboring amino acids, angles between three neighboring amino acids, and nonbonded interactions between amino acids that are separated by more than three bonds, respectively.

The bonding potential $U_{bond}=\sum_{i}V_{b}(r_{i,i+1})$, where i is the bead and

(4) $$V_{b}(r_{i,i+1})=\frac{k_{b}}{2}(r_{i,i+1}-r_{0}{)}^{2}.$$

We used $k_{b}=1000{kJmol}^{-1}{nm}^{-2}$ and $r_{0}=0.38\;nm$. The angular potential $U_{angle}=\sum_{i}V_{a}(\theta_{i})$ with

(5) $$V_{a}(\theta_{i})=\frac{k_{a}}{2}(\theta_{i}-\theta_{0}{)}^{2}.$$

$\theta_{i}$ is the angle formed between the three consecutive beads *i*, i+1, and *i* + 2 kJ mol^−1^ deg^−2^, and $\theta_{0}=127$° . The contact potential, $U_{contact}=\sum_{ij}V_{nb}(r_{ij},\epsilon_{IJ})$. The pairwise potential is a combination of excluded volume and contact terms given by

(6) $$V_{nb}(r_{ij})=\frac{|\epsilon |\sigma^{12}}{r_{ij}^{12}}+\epsilon C(r_{ij}).$$

i and j correspond to interacting beads, ϵ=−2.327339 kJ mol^−1^, and σ=0.586 nm.

In this force field, we placed 40 polymers in a 75 nm × 75 nm × 75 nm simulation box. After steepest decent energy minimization, we performed an NPT simulation for 0.1 s at 150 K and 1 bar, using a Parrinello–Rahman isotropic bariostat and time coupling constant of 1 ps. This resulted in a configuration of forty homopolymers compressed into a 6.3782 nm × 6.3782 nm × 6.3782 nm simulation box.

### MOFF simulations

From the starting configuration produced by the homopolymer simulation, we wanted to relax the contacts to be representative of those with ELPs before we began our explicit solvent simulations. For this equilibration step, we turned to the MOFF force field. ELPs are known to have minimal secondary structure in both the dense and dilute state (Rauscher and Pomès, 2017; Reichheld et al., 2017), so we assumed the secondary structure to be similar to that of a random coil. Under these simplifications, MOFF can be specified as

(7) $$U_{hom}(r)=U_{bond}+U_{angle}+U_{electrostatics}+U_{contact},$$

where $U_{bond}$ is described by Equation 4, $U_{angle}$ is described by Equation 5, $U_{electrostatics}$ accounts for electrostatic interactions between charged amino acids, and $U_{contact}$ accounts for non-bonded interactions.

$U_{electrostatics}$ accounts for electrostatic interactions between charged residues. Based on the Debye–Hückel theory, it can be approximated as

(8) $$U_{electrostatics}=\sum_{i,j}\frac{q_{i}q_{j}}{4\pi \epsilon_{0}r_{ij}\epsilon (r_{ij})}\exp(-r_{ij}/\lambda_{D}),$$

where $\epsilon_{0}$ is the permittivity of free space, $\lambda_{D}$ is the Debye screening length, $r_{ij}$ is the distance between particles i and j, and $q_{i}$ and $q_{j}$ are charges of particles i and j. Charges of individual residues can be found in Table S1 of Latham and Zhang, 2021. We used a distance-dependent dielectric constant, $\epsilon (r_{ij})$, to capture the change in the solvation environment upon protein folding (Mazur and Jernigan, 1991; Mehler and Solmajer, 1991). This dielectric takes the form

(9) $$\epsilon (r_{ij})=A+\frac{B}{1+ke^{-\lambda Br_{ij}}},$$

where A=−8.5525, k=7.7839, $\lambda =0.03627{nm}^{-1}$, and $B=\epsilon_{w}-A$, where $\epsilon_{w}=78.4$ is the dielectric constant of water.

The contact potential, $U_{contact}=\sum_{ij}V_{nb}(r_{ij},\epsilon_{IJ})$. The pairwise potential is a combination of excluded volume and contact terms given by

(10) $$V_{nb}(r_{ij},\epsilon_{IJ})=\frac{|\epsilon_{IJ}|\sigma_{IJ}^{12}}{r_{ij}^{12}}+\epsilon_{IJ}C(r_{ij}).$$

*I* and *J* correspond to the amino acid type of bead *i* and *j* is defined using the individual size of each amino acid type, and full parameters for the implementation of this potential can be found in Table S7 and Table S8 of Latham and Zhang, 2021.

In this force field, we conducted energy minimization, and then a 0.1 μs NVT simulation, with a timestep of 10 fs. During this simulation, we utilize the GROMACS simulated annealing protocol to linearly raise the temperature from 150 K to 300 K, with a time coupling constant of 100 ps.

### MARTINI simulations

The final implicit solvent, coarse-grained MOFF configuration was used as a starting point for our explicit solvent coarse-grained simulations with the MARTINI force field. To convert our α-carbon only configuration to a MARTINI representation, we converted the α-carbon snapshot to an all-atom representation using tleap, which is part of AMBER tools 2020 (Case, 2020). Next, we converted the all- atom configuration to a MARTINI3 configuration using martinize2 (Souza et al., 2021). We used random coil secondary structure throughout the entire peptide and enforced neutral termini for each peptide chain. We then expanded the Z-dimension of the simulation box to 40 nm and centered the protein in the simulation box. All simulations thus started with box dimensions of 6.3782 nm × 6.3782 nm × 40.0000 nm. This change in box dimensions was preparation for slab simulations, which enabled us to probe the physical properties of the condensate (Dignon et al., 2018b). In this setup, the high-density protein phase became a dense slab that was periodic in x and y, but did not interact with its periodic image in z due to the size of the box. The lack of periodic interactions in the z direction allowed the formation of two interfaces between a dense biopolymer phase and the solvent phase. We then solvated the peptide with MARTINI water and 1 M NaCl.

We started with steepest decent energy minimization. Then, we performed NVT equilibration with a timestep of 10 fs for a total simulation time of 5 ns. The protein and solvent were held at 313.15 K by separately coupling them to a velocity-rescaling thermostat with a time coupling constant of 1 ps. We then equilibrated our simulations in an NPT ensemble for 10 ns with a timestep of 20 fs. We separately coupled protein and solvent to a temperature of 313.15 K with a velocity-rescaling thermostat with a time coupling constant of 1 ps. The pressure was fixed using a Berendsen bariostat with a reference pressure of 1 bar, compressibility of 4.5 × 10^−5^ bar^−1^, and a time coupling constant of 5 ps. We then performed our production simulation in an NPT ensemble with semi-isotropic pressure coupling, which is sometimes called an NP_N_AT ensemble (Zhang et al., 1995). This technique fixes the pressure normal to the protein surface, which allows us to calculate the surface tension of the protein-solvent interface. Temperature was coupled separately to 313.15 K for the protein and solvent using a velocity-rescaling thermostat with a time coupling constant of 1 ps. A Parrinello–Rahman semi-isotropic bariostat was used to couple pressure at a time coupling constant of 10 ps. A compressibility of 4.5 × 10^−5^ bar^−1^ and pressure of 1 bar was used in the Z-dimension, but the compressibility was set to 0 in the X–Y dimensions. The production simulations lasted for 100 μs and used a timestep of 20 fs. The first 25 μs was discarded for equilibration, and configurations were recorded every 1000 ps for analysis.

Finally, not every α-carbon, implicit solvent representation from MOFF could be stably converted into a higher resolution, explicit solvent MARTINI representation. If energy minimization was unable to resolve clashes within the MARTINI configuration we converted from the final step in the MOFF simulation, we iteratively moved back one snapshot and repeated the procedure until a stable configuration was found.

We note that many studies of IDPs with MARTINI have scaled the protein-water interactions to improve agreement between simulation and experiment (Thomasen et al., 2022; Benayad et al., 2021). However, in the case of ELPs studied here, we find the surface tensions given by the default MARTINI model are reasonable based on experimentally informed theories of ELP condensates (Hassouneh et al., 2015) and experimental observations of surface tension in other biological condensates (Taylor et al., 2016), which suggest biological condensates may have interfacial tensions as large as ∼1 mN/m.

### Atomistic simulations

Proteins were described using the CHARMM36m force field (Huang et al., 2017). Proteins were capped using acetylated N-terminus and amidated C-terminus. TIP3P water was used, and the water-hydrogen Lennard–Jones well depth was scaled to –0.10 kcal/mol. This scaling has been shown to better describe IDPs in previous work (Huang et al., 2017).

All-atom simulations were started based on the final timestep of MARTINI simulations. MARTINI configurations were converted to CHARMM36m configurations using the backward script (Hofmann et al., 2012; Wassenaar et al., 2014). This code converts MARTINI configurations into all-atom representations and then ensures configuration stability through a series of short energy minimization and restrained molecular dynamics simulations. The final output for these configurations was utilized as the starting point for all-atom simulations. If the backward script did not result in as stable all-atom representation, we iteratively moved back one snapshot in the MARTINI and repeated the backward procedure until a stable all-atom initial configuration was found. We performed an additional round of energy minimization, and then equilibrated the protein in the NVT ensemble for 100 ps with a timestep of 1 fs. We used a Nose–Hoover thermostat to separately couple the protein and solvent to 313.15 K with a time coupling constant of 1.0 ps. Position restraints were applied with a force constant of 400 kJ mol^−1^ nm^−2^ for the backbone and 40 kJ mol^−1^ nm^−2^ for the side chains. We then performed NPT equilibration for 5 ns with a timestep of 2 fs. We used a Nose–Hoover thermostat to separately couple the protein and solvent to 313.15 K with a time coupling constant of 1.0 ps, and coupled the pressure using an isotropic Parrinello–Rahman bariostat with a compressibility of 4.5 × 10^−5^ bar^−1^, a reference pressure of 1 bar, and a time coupling constant of 2 ps. We then performed an additional equilibration in the NP_N_AT ensemble by using a semiisotropic bariostat for 5 ns with a timestep of 2 fs. We used a Nose–Hoover thermostat to separately couple the protein and solvent to 313.15 K with a time coupling constant of 1.0 ps. We coupled the pressure using an semiisotropic Parrinello–Rahman bariostat with a compressibility of 4.5 × 10^−5^ bar^−1^ and a reference pressure of 1 bar in the Z-dimension, but set the compressibility to 0 in the X–Y dimensions. The pressure-time coupling constant was 2 ps. We then performed production runs in the NP_N_AT ensemble. Like the previous simulation, we used a Nose–Hoover thermostat to separately couple the protein and solvent to 313.15 K with a time coupling constant of 1.0 ps, and coupled the pressure using an semiisotropic Parrinello–Rahman bariostat with a compressibility of 4.5 × 10^−5^ bar^−1^ and a reference pressure of 1 bar in the Z-dimension, but set the compressibility to 0 in the X–Y dimensions. The pressure-time coupling constant was 2 ps. Simulations lasted for 250 ns, and the first 50 ns was discarded for equilibration. We recorded timesteps every 10 ps for analysis.

### Monomer simulation details

In addition to the condensates, we carried out atomistic simulations of ELP monomers for comparison. These simulations were conducted on all 20 sequences and began from RosettaFold predictions of the peptide structure (Baek et al., 2021; Bonneau et al., 2001). RoseTTAFold was used in all cases except for V_5_M_5_, where not enough homologs were available for a prediction (Baek et al., 2021). In this case, Rosetta ab initio predictions were used instead (Bonneau et al., 2001). The force field was identical to the condensate simulations, and thus used the CHARMM36m force field, with shifted water potentials and capped termini (Huang et al., 2017). Each peptide was placed in a simulation box of 8 nm × 8 nm × 8 nm, which gives a >99% chance of the side of the simulation box being longer than the end-to-end distance of an ideal chain with a Kuhn length of 0.55 nm, as has been suggested for IDPs (Dignon et al., 2018a). Peptides were solvated in water with an ionic strength of 1 M.

After solvating each protein, we conducted energy minimization. Then, we performed 100 ps of equilibration with a timestep of 1 fs in the NVT ensemble. We used a Nose–Hoover thermostat to separately couple the protein and solvent to 313.15 K with a time coupling constant of 1.0 ps. Position restraints were applied with a force constant of 400 kJ mol^−1^ nm^−2^ for the backbone and 40 kJ mol^−1^ nm^−2^ for the side chains. Next, we performed NPT equilibration for 5 ns with a timestep of 2 fs. Simulations were conducted at 313.15 K and 1.0 bar. Finally, we conducted a production simulation in the NPT ensemble for 1 μs with a timestep of 2 fs, at 313.15 K and 1.0 bar. We excluded the first 100 ns of this production run for additional equilibration, which left 900 ns for analysis.

### Experimental characterization of ELP condensates

#### Plasmid synthesis

Cloning vector pET-28a+was modified to contain endonuclease recognition sites of BseRI, AcuI, and BglI for inserting the designed ELP sequence, similar to McDaniel et al., 2010. Modified pET-28a+ was linearized using BseRI (NEB, China) and dephosphorylated with rSAP (NEB) for ELP assembly. ssDNA encoding for five pentapeptides (5-mer) sequences was ordered through GeneWiz, China. Then the ssDNA chains were annealed and phosphorylated to form double-stranded duplexes with sticky overhangs. Linearized pET-28a+ vectors and duplexes were ligated by the T4 DNA ligase (NEB). After obtaining 5-mer ELP plasmids, we performed the iterative cloning steps to increase the repeats through recursive directional ligation by the plasmid reconstruction method (McDaniel et al., 2010) until achieving the target length of ELP. The sequences of all plasmids created at each step were confirmed by DNA sequencing.

### Protein expression and purification

The targeted ELP plasmids were transformed into *Escherichia coli* BL21 (DE3) cells. Cells were incubated overnight on the LB plate with kanamycin. On the second day, a single colony was inoculated into 5 mL Luria Broth (LB) and serially diluted (1:100) into four different starter flasks (A–D). Starter cultures were shaken for 12–14 hours at 37 °C, and the culture with the OD600 0.6–0.8 was inoculated into 1 L Terrific Broth (TB) containing 50 μg/mL kanamycin at 1:100 dilution. This culture was grown at 37 °C and 220 rpm until the media OD600 reached 0.8, and the culture was then induced to express the protein with 0.5 mM isopropyl-β-d-thiogalactoside (IPTG). The cultures were then kept shaking for 20–24 hours at 37°C and 200 rpm before harvesting. The cells were collected by centrifuging at 6000 rpm at 4 °C for 10 min and resuspended in 1× PBS buffer (pH 7.4). The proteins were released from the cells by sonicating on ice for 6 min, with 2 s of pulsing followed by 8 s of resting on ice. The lysate was centrifuge at 14,000 rpm at 4°C for 40 min. The supernatant was then collected and mixed with polyethyleneimine to a final concentration of 0.5 w/v% to precipitate nucleic acids. The mixture was then centrifuged at 14,000 rpm and 4°C for 40 min. The supernatant was collected and added with NaCl(s) to get the final concentration of 4.5 M NaCl. The mixture was then shaken at 37°C to precipitate ELP and centrifuged at 10,000 rpm at 37°C for 40 min. The pellet was saved and resuspended in double-distilled H_2_O. This mixture was then shaken on ice to dissolve the ELP. The inverse transition cycling was repeated 1–2 more times (Meyer and Chilkoti, 1999). The protein solution was dialyzed in a 10 kDa membrane (SnakeSkin, Thermo Fischer Scientific) against 2 L double-distilled H_2_O at 4°C, changing the dialysis water one time. The protein purity was determined by 12% SDS-PAGE gel electrophoresis and stained with 0.5 M copper chloride. ELP proteins were then concentrated and lyophilized to remove the water.

### Sequence dependence of microviscosity and polarity

All ELP sequences were expressed in bacteria *E. coli* and purified to more than 95% purity, as analyzed by SDS-PAGE (Appendix 1—figure 4). The purified ELPs were subjected to mass spectroscopy measurement to confirm their identity (Appendix 1—table 2 and Appendix 1—figure 5). Using turbidity as a signal to report on the formation of liquid droplets as a function of temperature change, we found that V_30_A_30_ and A_30_V_30_ exhibited the same $T_{t}$ (transition temperature for phase separation) values (Figure 3—figure supplement 1A and B), suggesting that both sequences underwent almost identical intermolecular interactions to transit from the one-liquid diffusive phase to the two-liquid phase separated phase. The same is true for V_30_G_30_ and G_30_V_30_ (Figure 3—figure supplement 1C and D). To measure the viscosity and polarity of different micro-organizations of blocks, we used the established microenvironment-sensitive fluorogenic probes: boron dipyrromethene (BODIPY) and 7-sulfonamide benzoxadiazole (SBD). Based on previous reports, the fluorescence lifetime (τ) of BODIPY would increase with elevating microviscosity, whereas the τ of SBD would rise with decreasing micro-polarity. The correlation of their τ values can be quantitatively determined using standard solvents, thus providing equations to quantify viscosity (η) and polarity (as ϵ, dielectric constants) via the measurement of τ values (Appendix 1—figure 6).

**Appendix 1—table 1.** Maximum allowed solvent-accessible surface area (SASA) for each amino acid, taken from Tien et al., 2013.

Amino acid	Maximum SASA (Å^2^)
ALA	129
ARG	274
ASN	195
ASP	193
CYS	167
GLN	225
GLU	223
GLY	104
HIS	224
ILE	197
LEU	201
LYS	236
MET	224
PHE	240
PRO	159
SER	155
THR	172
TRP	285
TYR	263
VAL	174

**Appendix 1—table 2.** Mass spectrum of purified ELP.

	Mass (Da)	
	Theoretical	MALDI-MS
V_30_A_30_	24097.04	23898.62
A_30_V_30_	24097.04	23916.19
V_30_G_30_	23676.23	23495.55
G_30_V_30_	23676.23	23498.98

Since we used V_30_X_30_ and X_30_V_30_ to quantify the V- and X-end of the V-X blocks, it is possible that the observed differences arose from the innate property of the V_30_X_30_ and X_30_V_30_ sequences. To rule out this artifact, we formed the ELP condensates with sequences of V_30_X_30_, X_30_V_30_, or the V_30_X_30_ and X_30_V_30_ mixture. The condensates were subsequently treated with the aldehyde-BODIPY and methyl-ester SBD fluorophores without the NHS ester reactive warhead (Figure 3—figure supplement 2). After brief incubation, aldehyde-BODIPY and methyl-ester SBD fluorophores were recruited into and homogeneously distributed in the ELP condensates. The fluorescence lifetime of aldehyde-BODIPY was the same for V_30_A_30_ (4.96 ns), A_30_V_30_ (4.99 ns), and their mixture (4.98 ns) (Figure 3—figure supplement 2B, upper panel). Interestingly, this value is around the average (4.89 ns) of the A-end (4.35 ns) and the V-end (5.43 ns)-labeled NHS-BODIPY. For the SBD measurement, methyl-ester SBD resulted in almost identical lifetime values of V_30_A_30_ (8.25 ns), A_30_V_30_ (8.27 ns), and their mixture (8.28 ns) (Figure 3—figure supplement 2B, lower panel), again around the average values (7.88 ns) of the A-end (7.00 ns) and the V-end (8.75 ns)-labeled NHS-SBD. In addition to the V-A blocks, similar observations were made for the V-G blocks as V_30_G_30_ and G_30_V_30_ sequences (Figure 3—figure supplement 2C). The slight difference between the results is attributed to the experiment errors. Because the fluorophores did not covalently label the amino-terminus of the ELP peptides, their lifetime reports closer to the averaged property of the condensates instead of the microscopic property of the V-end or the X-end when the number of molecules is sufficient and the molecular distribution has no preference. Our results reveal that the V_30_X_30_ and X_30_V_30_ condensates exhibited similar macroscopic viscosity or polarity, suggesting that the previously observed different viscosity or polarity of V_30_X_30_ and X_30_V_30_ could be attributed to the microscopic property of the V-end or X-end.

### Supplemental theory

#### Relationship between the transition temperature $T_{t}$ and the critical temperature $T_{C}$

The temperature measured by Urry et al. and presented in the main text corresponds to the transition temperature, $T_{t}$ (Urry, 1997). This temperature is defined as the value above which ELP forms an insoluble coacervate phase. The value of $T_{t}$ depends on the concentration of the polymer solution.

As shown by Chilkoti and coworkers (Meyer and Chilkoti, 1999; Meyer and Chilkoti, 2004), the critical temperature $T_{c}$ is indeed linearly related to $T_{t}$ with the following relationship:

(11) $$T_{t}=T_{c}+\frac{k}{\text{length}}\ln\frac{C_{c}}{\text{conc}}.$$

The above equation highlights the dependence of $T_{t}$ on the chain length (length) and polymer concentration (conc). The parameter $C_{c}$ is the corresponding theoretical polypeptide concentration that would be required to achieve $T_{c}$ and k is the proportionality constant.

#### Relationship between the critical temperature $T_{c}$, the Flory–Huggins parameter χ, and the surface tension, τ

The Flory–Huggins parameter, χ, is defined as

(12) $$\chi =\frac{z}{2k_{B}T}[\epsilon_{\text{pp}}+\epsilon_{\text{ss}}-2\epsilon_{\text{ps}}]=\frac{z\Delta \epsilon }{k_{B}T},$$

where $\Delta \epsilon =\frac{1}{2}[\epsilon_{\text{pp}}+\epsilon_{\text{ss}}-2\epsilon_{\text{ps}}]$ is the coordination number, T is the temperature, $k_{B}$ is the Boltzmann constant, and $\epsilon_{\text{pp}}$ are the strength of polymer-polymer, solvent-solvent, and polymer-solvent interactions respectively (Flory, 1942).

From the original derivation of Flory–Huggins theory, it can be shown that phase separation occurs when χ is greater than its critical value, or $\chi >\chi_{c}\approx \frac{1}{2}$. From $\chi_{c}$, we can derive the critical temperature as

(13) $$T_{c}=\frac{2z\Delta \epsilon }{k_{B}}.$$

Therefore, $\chi =\frac{T_{c}}{2T}$

Relationships between the Flory–Huggins parameter, χ, and interfacial tension (τ) have been investigated, and the relationship can be approximated as

(14) $$\tau \propto \chi^{\alpha },$$

where α is a positive constant, whose exact value depends on the proximity of χ to the critical value of χ necessary for phase separation ($\chi_{C}$) (Roe, 1975; Helfand and Tagami, 1972). Correspondingly, we have $\tau \propto T_{c}^{\alpha }$.

We note that the expression for χ (Equation 12) is simplified and assumes that Δϵ is independent of temperature. However, changes in solvent packing upon phase separation can result in entropic contributions Rubinstein and Colby, 2003. In general, Δϵ should be expressed as Δϵ=Δh+TΔs. The entropic contributions are essential for giving rise to phase separations with lower critical temperatures. Therefore, in the generalized case, we have

(15) $$\chi =\frac{z\Delta \epsilon }{k_{B}T}=\frac{z(\Delta h+T\Delta s)}{k_{B}T}.$$

At the critical temperature, we have

(16) $$\frac{1}{2}=\frac{z(\Delta h+T_{C}\Delta s)}{k_{B}T_{C}},$$

and

(17) $$T_{C}=\frac{z\Delta h}{k_{B}/2-z\Delta s}.$$

For systems exhibiting lower critical solution temperatures (LCST), Δh<0 and Δs>0. For $T_{c}>0$, we have $k_{B}/2<z\Delta s$.

Plugging the above expression back to Equation 15, we have

(18) $$\chi =\frac{k_{B}T_{C}/2-z\Delta sT_{C}+zT\Delta s}{k_{B}T}=\frac{T_{C}}{T}\left(\frac{1}{2}-\frac{z\Delta s}{k_{B}}\right)+\frac{z\Delta s}{k_{B}}.$$

Furthermore, as shown by Schauperl et al., 2016, Δs, while significant, remains relatively constant across different amino acids. Correspondingly, we have $\tau \propto {\left[\frac{T_{c}}{T}\left(\frac{1}{2}-\frac{z\Delta s}{k_{B}}\right)+\frac{z\Delta s}{k_{B}}\right]}^{\alpha }$ holds true in the more general case as well. Since $\frac{z\Delta s}{k_{B}}>\frac{1}{2}$, the slope between τ and $T_{C}$ is negative.

### Relationship between the critical temperature, $T_{c}$ and hydrophobicity scales

As shown in Equation 13, in the simple case $T_{c}$ is linearly related to Δϵ. For the more general case, from Equation 17, we have

(19) $$T_{C}=\frac{z\Delta h}{k_{B}/2-z\Delta s}=\frac{z\Delta \epsilon }{k_{B}/2-z\Delta s}-\frac{zT\Delta s}{k_{B}/2-z\Delta s}.$$

Assuming that Δs is relatively independent of amino acids, as shown by Schauperl et al., 2016, $T_{c}$ is again linearly proportional to Δϵ.

$\Delta \epsilon =\frac{1}{2}[\epsilon_{\text{pp}}+\epsilon_{\text{ss}}-2\epsilon_{\text{ps}}]$ can indeed be interpreted as the free energy cost of transferring a polymer bead from a solution phase to a polymer phase. It corresponds to the change of energy from a mixed state, with contacts between polymer and solvent ($\epsilon_{\text{ps}}$), to the demixed state with only polymer-polymer ($\epsilon_{\text{pp}}$) and solvent-solvent ($\epsilon_{\text{ss}}$) contacts.

Therefore, the transfer free energy, and the interactions among amino acids of ELPs, are expected to correlate with the critical temperature.

### Experimental sequences

V_30_A_30_
M-(GVGVP)_30_-(GAGVP)_30_-GY
A_30_V_30_
M-(GAGVP)_30_-(GVGVP)_30_-GY
V_30_G_30_
M-(GVGVP)_30_-(GGGVP)_30_-GY
G_30_V_30_
M-(GGGVP)_30_-(GVGVP)_30_-GY
