# Supplementary figures and images for: Microphase separation produces interfacial environment within diblock biomolecular condensates
Source: eLife. 2025 Mar 26;12:RP90750. doi: 10.7554/eLife.90750 (PMC11942181; doi:10.7554/eLife.90750)

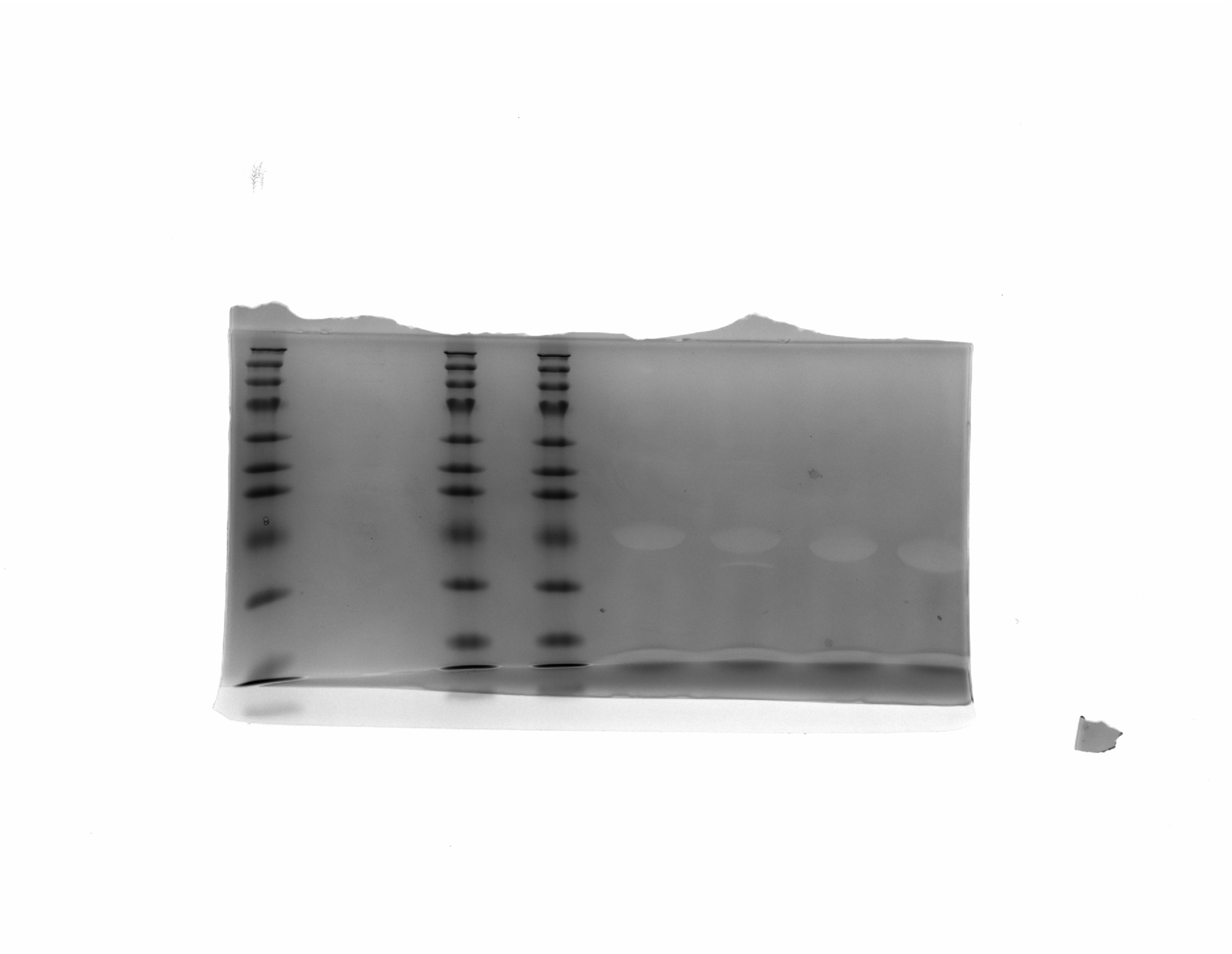

Supplement: Appendix 1—figure 4—source data 1. [file elife-90750-app1-fig4-data1.zip › Appendix-Figure 4 Source Data - 1.png]

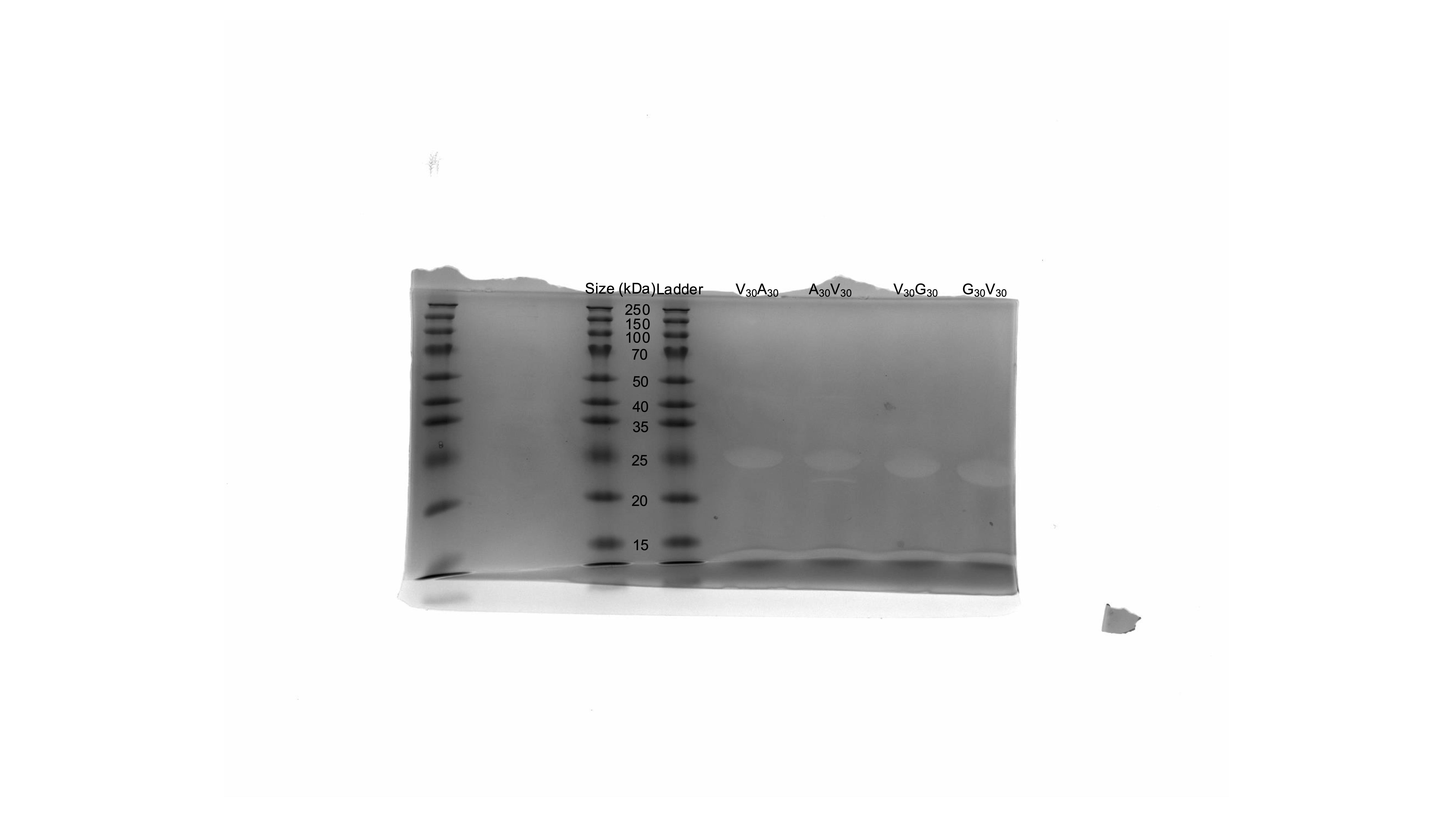

Supplement: Appendix 1—figure 4—source data 2. [file elife-90750-app1-fig4-data2.zip › Appendix-Figure 4 Source Data 2.jpg]
