# Supplementary material for: Microphase separation produces interfacial environment within diblock biomolecular condensates
Source: eLife. 2025 Mar 26;12:RP90750. doi: 10.7554/eLife.90750 (PMC11942181; doi:10.7554/eLife.90750)
Supplement: Peer review history

# Editor report: eLife assessment

**DOI:** 10.7554/eLife.90750.4.sa0

**Authors:** Rosana Collepardo

**Affiliations:** University of Cambridge United Kingdom

**Keywords:** Compelling, Important

This **important** study investigates the structural organization of a series of diblock elastin-like polypeptide condensates. The methodology is highly **compelling** as it combines multiscale simulations and fluorescence lifetime imaging microscopy experiments. The results increase our understanding of model biomolecular condensates.

# Referee report: Reviewer #1 (Public Review)

**DOI:** 10.7554/eLife.90750.4.sa1

This is an interesting, informative, and well-designed study that combines theoretical and experimental methodologies to tackle the phenomenon of higher-resolution structures/substructures in model biomolecular condensates.

The authors have adequately addressed my previous concerns.

# Referee report: Reviewer #2 (Public Review)

**DOI:** 10.7554/eLife.90750.4.sa2

Summary:

Latham A.P. et al. apply simulations and FLIM to analyse several di-block elastin-like polypetides and connect their sequence to the micro-structure of coacervates resulting from their phase-separation.

Strengths:

Understanding the molecular grammar of phase separating proteins and the connection with mesoscale properties of the coacervates is highly relevant. This work provides insights into micro-structures of coacervates resulting from di-block polypetides.

Weaknesses:

The results apply to a very specific architecture (di-block polypetides) with specific sequences.

# Author comment: Author response

**DOI:** 10.7554/eLife.90750.4.sa3

**Authors:** Andrew P Latham, Longchen ZHU, Dina A Sharon, Songtao YE, Adam P Willard, Xin Zhang, Bin Zhang

**Affiliations:** Massachusetts Institute of Technology Cambridge United States; Westlake University Hangzhou China; Massachusetts Institute of Technology Cambridge United States; Westlake University Hangzhou China; Massachusetts Institute of Technology Cambridge United States; Westlake University Hangzhou China; Massachusetts Institute of Technology Cambridge United States

The following is the authors’ response to the previous reviews.

The authors have addressed my comments. As a final minor point, regarding comment 2,these condensates are likely viscoelastic rather than purely viscous. It is prudent toindicate that the data may refer to an apparent viscosity.

We added the following text to the manuscript to highlight the viscoelastic nature of ELPcondensates, and the relationship of reported values with the steady state viscosity.“It is worth noting that the reported values, although related, may not quantitativelyrepresent the steady-state viscosity. This discrepancy arises from the slow relaxationtimescale inherent in ELP condensates with viscoelastic properties.”
